# How mathematical models might predict desertification from global warming and dust pollutants

**DOI:** 10.1016/j.mex.2025.103259

**Published:** 2025-03-10

**Authors:** Eman Hakeem, Shireen Jawad, Ali Hasan Ali, Mohamed Kallel, Husam A. Neamah

**Affiliations:** a Department of Mathematics, College of Science, University of Baghdad, Baghdad, Iraq; b Institute of Mathematics, University of Debrecen, Pf. 400, H-4002 Debrecen, Hungary; c Jadara Research Center, Jadara University, Irbid 21110, Jordan; d Department of Physics, College of Science, Northern Border University, Arar, Saudi Arabia; e Mechatronics Department, Faculty of Engineering, University of Debrecen, Ótemető u. 4-5, Debrecen, 4028, Hungary

**Keywords:** Mathematical model, Equilibrium points, Stability analysis, Desertification, Dust pollutants, Plant biomass, Global warming, Stability analysis, The 4th-order Runge-Kutta method approximation

## Abstract

Global warming and dust pollutants endanger humans and the ecosystem. One very efficient way to reduce emissions of greenhouse gases and dust is to use plant biomass in a greenbelt. This study provides a mathematical model for how dust pollutants and climate change affect plant biomass dynamics. The proposed model is thoroughly described. The model's analysis is centered on identifying prospective equilibrium positions. The study indicates that it is feasible to establish two steady states. The stability analysis illustrates that both steady states are consistently stable under the specified conditions. The local bifurcations at each steady state are derived; specifically, transcritical bifurcation may occur if a plant's growth rate is selected as a bifurcation point. The theoretical study is validated through numerical simulations. Desertification may arise if the intrinsic growth rate of plant biomass, the dust pollutants-induced plant biomass depletion coefficient, and the coefficient of natural depletion of dust contaminants are not effectively managed, according to the numerical simulation result.

• This research describes how to make a nonlinear model and sets its parameters to simulate the risk of desertification caused by global warming and dust pollutants.
• The proposed model's behaviour is described using stability analysis theory as a methodology.
• Numerical simulations confirm the performance of the proposed methodology.

## Graphical abstract

**Figure:**
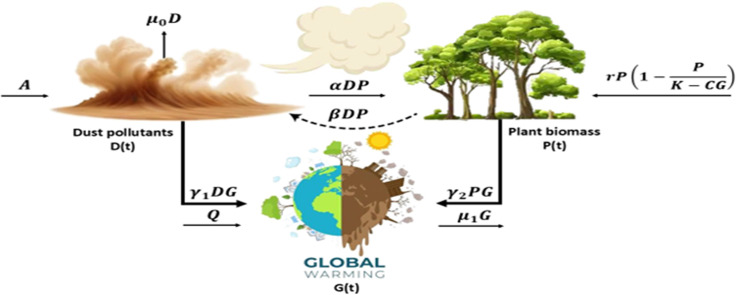

Specifications table

**Table:** 

Subject area:	Mathematics and Statistics
More specific subject area:	Ecosystem, Stability analysis Numerical Analysis
Name of your method:	Stability analysis, The 4th-order Runge-Kutta method approximation
Name and reference of original method:	Jackiewicz, Z., & Tracogna, S. (1995). A general class of two-step Runge–Kutta methods for ordinary differential equations. *SIAM Journal on Numerical Analysis, 32*(5), 1390–1427. Link: https://epubs.siam.org/doi/abs/10.1137/0732064
Resource availability:	Sundar, S., & Naresh, R. (2017). Modeling the effect of dust pollutants on plant biomass and their abatement from the near earth atmosphere. *Modeling Earth Systems and Environment, 3*, 1–13. Link: https://link.springer.com/article/10.1007/s40808-017-0302-3

## Background

Disease transmission estimates, future population projections, and other scientific and physical phenomena can be better understood and described using mathematical models [1,[2], [3], [4]]. Plant biomass is significantly impacted by global warming because the conditions that plants need to grow and thrive are altered by rising temperatures, rising atmospheric CO₂ levels, and shifting precipitation patterns. The total mass of live plant matter in an ecosystem is known as plant biomass, and it is essential for biodiversity, soil health, and carbon sequestration. Stress induced by global warming may affect a plant's capacity to survive, develop, and produce [5]. Global warming may disrupt plant communities in the long term, with certain species flourishing while others struggle. Changes in the distribution and abundance of plant biomass could significantly impact ecosystems, including decreased food availability for herbivores, biodiversity shifts and altered carbon storage [6,7].

Global warming is recognized as one of the primary contributors to desertification, as the persistent rise in temperatures results in altered weather patterns and reduced precipitation, which have a detrimental impact on the vegetation cover in verdant areas. As temperatures rise and arid periods lengthen, plants become more susceptible to drought and stress, decreasing their growth, soil degradation, and fertility loss. In addition, the distribution of groundwater and surface water is disrupted by changing weather patterns as drought periods become more severe and prolonged in numerous previously verdant regions. This results in land degradation and the progressive transformation of these areas into deserts [8].

Dust pollutants adversely impact vegetation since dust particles and other airborne contaminants compromise the health and development of plants. One of the most important impacts on plants is the impediment to photosynthesis: Accumulated dust on plant foliage obstructs sunlight, which is essential for photosynthesis, diminishing the plant's capacity to generate the sustenance and energy required for their growth. In addition, dust pollutants obstruct leaf stomata: Dust particles may obstruct leaf stomata, so restricting gas exchange, which induces stress in plants and impairs their uptake of vital gases, such as carbon dioxide, and the release of oxygen [9]. Furthermore, dust pollutants can contain harmful substances, including heavy metals and industrial contaminants, which may impair leaf structure and alter plant growth. Dust pollutants could alter soil properties through the accumulation of dust on soil, which can modify its physical and chemical characteristics, impairing the capacity of roots to absorb water and nutrients, hence detrimental to plant growth. These impacts result in a deterioration of vegetation quality and health, potentially harming agriculture and natural ecosystems, diminishing biodiversity, and heightening the risk of desertification [10]. Desertification refers to the deterioration of land in arid and semi-arid regions caused by natural factors like climate change and anthropogenic factors such as deforestation and unsustainable agricultural practices. Desertification results in diminished agricultural output, precipitating food scarcity and elevated poverty levels, particularly in rural areas reliant on agriculture. It also exacerbates mass migration to urban areas, placing strain on infrastructure and services. Environmentally, it results in less biodiversity, heightened sandstorms, and elevated temperatures due to the depletion of vegetative cover. It results in substantial government economic losses due to diminished agricultural output and the elevated expenses associated with combatting desertification and restoring damaged regions [8,9]. Mathematical modeling is vital in solving many life problems [[11], [12], [13], [14], [15], [16], [17], [18], [19], [20]]. Mathematical models of desertification, forest biomass, climate change, and dust pollutants are under-researched. For instance, Prabir has formulated a mathematical model that considers global warming and forest biomass as separate compartments. He has made the assumption that global warming influences the expansion of forest biomass. He discovered that the rate of global warming will be brought down if the area of forest biomass is increased [21]. Previous studies primarily concentrate on developing mathematical models for polluted environments. Dubey et al. [22] proposed a mathematical model to analyze the depletion of resource biomass in plants resulting from industrialization and pollution. It was observed that, in small periodic influxes of pollutants into the environment, the resource biomass exhibits periodic behaviour when the depletion rate coefficient of the environmental pollutant is low. However, resource biomass approaches equilibrium if this coefficient exceeds a threshold value. Further, Shyam et al. have considered the impact of particulate contaminants on plant biomass. Their model considered three variables: the density of plant biomass, the concentration of dust, and the density of water droplets. They have discovered that spraying water particles into the near-earth atmosphere is unnecessary when the concentration of dust contaminants is below its threshold concentration [23].

Therefore, in view of the above, there is a shortage of research on the influence of dust pollutants and global warming on the density of plant biomass. Hence, this investigation is focused on examining the impact of dust pollutants and global warming on the density of plant biomass. This research aims to investigate the dynamics of the dust pollutants– plant biomass-global warming model via a nonlinear mathematical model. Considering these effects, we propose a DPG model of dust pollutants– plant biomass-global warming interaction. This paper's findings provide additional context for Shyam et al. [23] by substituting the water spray equation with the one for global warming. This alteration enables us to precisely determine the important role that plant biomass plays in maintaining the balance of the ecosystem in the face of global warming and dust pollutants. Therefore, we believe it is essential to investigate this phenomenon, as it helps mitigate desertification.

## Method details

Using mathematical modelling, we endeavour to define the impact of global warming and dust pollutants on plant biomass dynamics in the present study. This study might be beneficial in evaluating the key parameters that affect, prevent or control the cause of desertification. Here is a concise summary of the paper's main objectives:

a) Recognize the causes of desertification and determine the management factors that may mitigate or prevent its emergence.
b) Explore the model's ability to predict and manage desertification by adopting various parameters.
c) Examine the well-posedness of our model's solutions by applying the Banach fixed point theorem.
d) Identify the potential equilibrium points and analyze their stability using the Routh-Hurwitz criterion.
e) Simulate the behaviour of model (1) using the 4th-order Runge-Kutta method approximation.

### Structural configuration

Let us examine a DPG system that puts forward the following hypothesis: dust pollutants D(t), plant biomass P(t), and global warming phenomena G(t). The modeling procedure has been predicated on the following assumptions:

1. Suppose that the rate of dust particulate emission into the atmosphere is A. The dust contaminants deplete naturally at a rate $\mu_{0}$ [24].
2. The plant biomass reduces the concentration of dust particles in the atmosphere, which functions as a dust scavenger. The decreasing concentration of dust particles is directly proportional to the amount of dust particles and the density of plant biomass (i.e. αDP), where α is a dust particle depletion rate coefficient [23].
3. Plant biomass is hypothesized to grow with the intrinsic growth rate r and carrying capacity k in the absence of the impact of global warming and dust pollutants.
4. It is assumed that desertification caused by climate change is considered a factor reducing carrying capacity by cg, where c accounts for a reduction rate in carrying capacity due to global warming phenomena [8,25].
5. Due to the increasing concentration of dust particles, it is presumed that the plant biomass is depleted (βDP), where β is a plant biomass depletion rate coefficient [23].
6. Various human activities (i.e. Q), including urbanization, industrialization, modern lifestyle, etc., contribute to the continuous increase in global warming's effect on the atmosphere and earth's surface [26,27].
7. Various studies indicate that global warming can be mitigated or reduced by implementing multiple control strategies, including reducing fuel consumption, plantation, etc. Therefore, we assume $\mu_{1}$ is global warming depletion owing to human control strategies [21], and $\gamma_{2}$, is the depletion rate of global warming due to plantation [28,29].
8. Various studies suggest that dust pollutants deflect part of the sun's rays before they reach the earth's surface, limiting the amount of heat the planet receives and producing a cooling effect. This impact may alleviate certain consequences of global warming [30]. Therefore, we assume $\gamma_{1}$ is the depletion rate of global warming due to the cooling effect of dust mass on the climate.

Based on the assumptions above, we have developed the following DPG system.:

(1) $$\begin{array}{c} \frac{dD}{dt}=A-\mu_{0}D-\alpha DP=F_{1}\left(D,P\right),\\ \frac{dP}{dt}=rP\left(1-\frac{P}{K-CG}\right)-\beta DP=F_{2}\left(D,P,G\right),\\ \frac{dG}{dt}=Q-\gamma_{1}DG-\gamma_{2}PG-\mu_{1}G=F_{3}\left(D,P,G\right), \end{array}$$

with the initial conditions D(0)≥0,P(0)≥0 and G(0)≥0. The parameters of the DPG model are delineated in Table 1 below.

**Table 1:** Description of the DPG system's parameters.

Parameters	Denotation	Values	Source
A	The rate of dust pollutants from diverse sources into the atmosphere.	10	[23]
$\mu_{0}$	The coefficient of natural depletion of dust pollutants.	0.1	[23]
$\alpha_{\;}$	Plant biomass-induced dust pollutants depletion coefficient.	0.01	[23]
r	The growth rate of plant biomass.	0.22	[23]
k	Plant biomass's carrying capacity.	30	[23]
c	Global warming-induced desertification.	0.01	[25]
β	Dust pollutants-induced plant biomass depletion coefficient.	0.001	[23]
Q	Factors contributing to the rising of global warming.	0.821	[25]
$\gamma_{1}$	The depletion rate coefficient of global warming due to dust pollutants.	0.001	Estimated
$\gamma_{2}$	The depletion rate coefficient of global warming due to plant biomass.	0.003	[21]
$\mu_{1}$	The depletion of global warming due to human interventions.	0.001	[21]

**Fig. 1:**
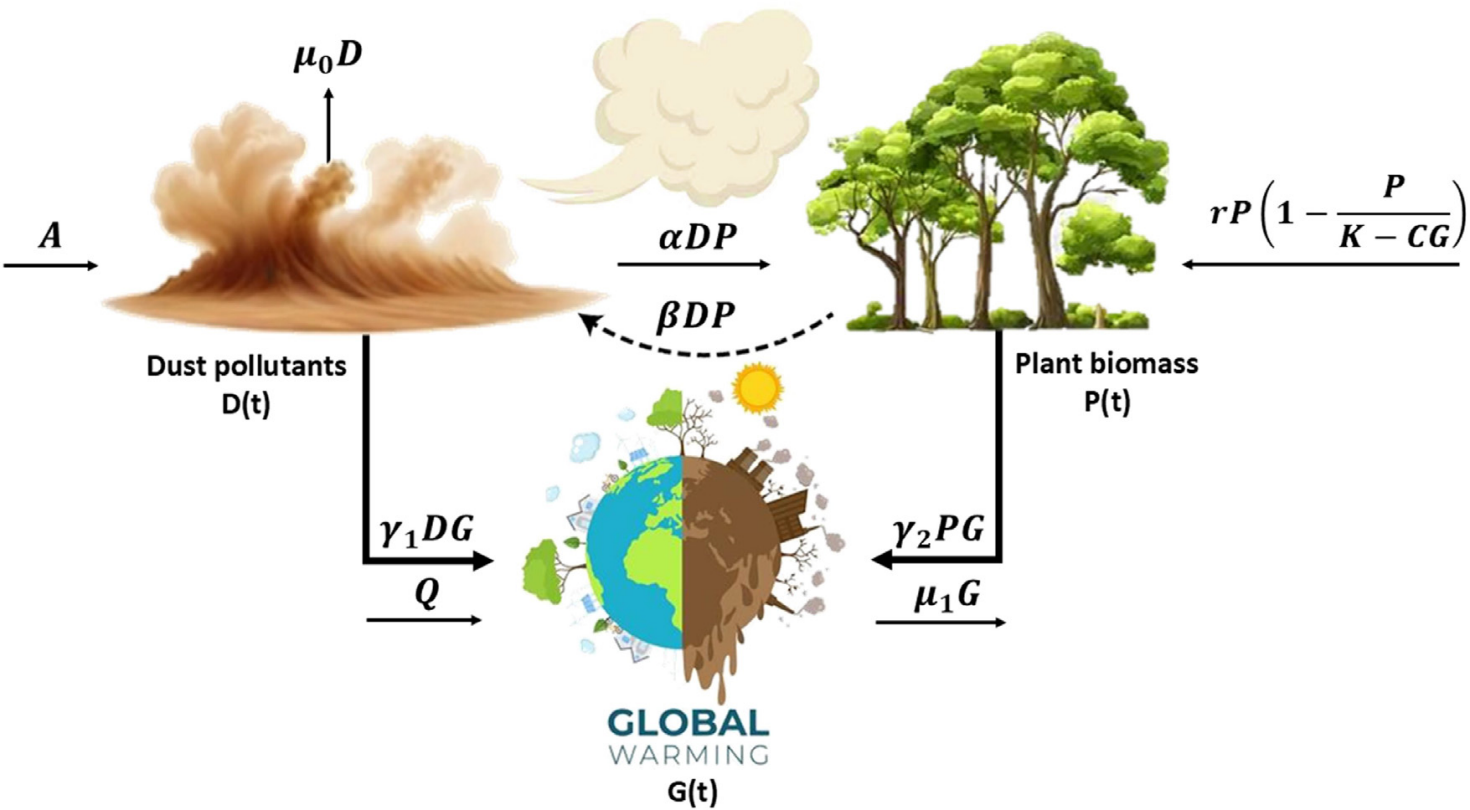
Schematic diagram of the DPG model.

In addition, the schematic sketch of the DPG model is explained in Fig. 1.

### Positivity and boundedness

The positivity and boundedness of all solutions of the DPG model in the positive orthant of $R_{+}^{3}$ are established by the subsequent theorems., We refer to [17,31,32] for a detailed argument.

Theorem 1 *All solutions*D(t),P(t),*and*G(t)*of the DPG system with the initial conditions*$\left(D(0),\;P(0),\;G(0)\right)\in R_{+}^{3}$*remains non-negative*.

*Proof*. Let D(t),P(t),and G(t) be the solution of the DPG system, with the initial condition $\left(D(0),\;P(0),\;G(0)\right)\in R_{+}^{3}$, we derive

$$P\left(t\right)=P\left(0\right)\exp\left\{\int_{0}^{t}\left[r-\frac{rP\left(\delta \right)}{k-cG\left(\delta \right)}-\beta D\left(\delta \right)\right]d\delta \right\}>0$$

From the dust pollutants equation of the DPG model, we obtain

$$dD=\left[A-D\left(\mu_{0}+\alpha p\right)\right]dt$$

Then, after substituting the formula of P(t) and eliminating the non-negative term yields

$$dD\geq \left[-D\left(\mu_{0}+\alpha P\left(0\right)\exp\left\{\int_{0}^{t}\left[r-\frac{rP\left(\delta \right)}{k-cG\left(\delta \right)}-\beta D\left(\delta \right)\right]d\delta \right\}\right)\right]dt.$$

Integrating the above equation for D(t) yields

$$D\left(t\right)\geq D\left(0\right)exp\left\{\int_{0}^{t}\left[-\mu_{0}-\alpha P\left(0\right)\exp\left\{\int_{0}^{t}\left[r-\frac{rP\left(\delta \right)}{k-cG\left(\delta \right)}-\beta D\left(\delta \right)\right]d\delta \right\}\right]d\delta \right\}>0.$$

From the global warming equation of the DPG system, we attain

$$dG=\left(Q-\gamma_{1}DG-\gamma_{2}PG-\mu_{1}G\right)dt$$

$$dG\geq -G\left(\gamma_{1}D\left(0\right)exp\left\{\int_{0}^{t}\left[\mu_{0}+\alpha P\left(0\right)\exp\left\{\int_{0}^{t}\left[r-\frac{rP\left(\delta \right)}{k-cG\left(\delta \right)}-\beta D\left(\delta \right)\right]d\delta \right\}\right]d\delta \right\}+\gamma_{2}P\left(0\right)\exp\left\{\int_{0}^{t}\left[r-\frac{rP\left(\delta \right)}{k-cG\left(\delta \right)}-\beta D\left(\delta \right)\right]d\delta \right\}+\mu_{1}\right)dt$$

By integrating the above equation, we obtain

$$\begin{array}{ccc} & & G\left(t\right)\geq G\left(0\right)exp\{\int_{0}^{t}-(\gamma_{1}D\left(0\right)exp\left\{\int_{0}^{t}\left[\mu_{0}+\alpha P\left(0\right)\exp\left\{\int_{0}^{t}\left[r-\frac{rP\left(\delta \right)}{k-cG\left(\delta \right)}-\beta D\left(\delta \right)\right]d\delta \right\}\right]d\delta \right\}\\ & & \;+\gamma_{2}P\left(0\right)\exp\left\{\int_{0}^{t}\left[r-\frac{rP\left(\delta \right)}{k-cG\left(\delta \right)}-\beta D\left(\delta \right)\right]d\delta \right\}+\mu_{1})d\delta \}>0. \end{array}$$

Therefore, any solution (D(t),P(t),G(t)) that starts in $R_{+}^{3}$ with the initial conditions (D(0),P(0),G(0)) will remain in $R_{+}^{3}$.

Theorem 2 *The DPG model's solutions are uniformly bounded*.

Proof: let $\left(D(0),\;P(0),\;G(0)\right)\in R_{+}^{3}$ be an initial condition for the DPG model. By applying the standard comparison theory [33] to both the first and third equations of the DPG model, it is obtained

$$\frac{dD}{dt}=A-\mu_{0}D-\alpha DP\leq A-\mu_{0}D\Rightarrow \lim_{t\to \infty }sup\left[D\left(t\right)\right]\leq \frac{A}{\mu_{0}}.$$

and

$$\frac{dG}{dt}=Q-\gamma_{1}DG-\gamma_{2}PG-\mu_{1}G\leq Q-\mu_{1}G\Rightarrow \lim_{t\to \infty }sup\left[G\left(t\right)\right]\leq \frac{Q}{\mu_{1}}=G_{m}.$$

From the plant biomass equation of the DPG system, we get

$$\frac{dP}{dt}=rP\left(1-\frac{P}{k-cG}\right)-\beta DP\leq rP\left(1-\frac{P}{k-cG}\right)\leq rP\left(1-\frac{P}{k-cG_{m}}\right)$$

Again, by the standard comparison method, we have

$$\lim_{t\to \infty }sup\left[p\left(t\right)\right]\leq k-cG_{m}$$

Therefore, the attracting region for the DPG model is

$$\psi =\left\{\left(D,\;P,\;G\right)\in R_{+}^{3}:\;D\left(t\right)\leq \frac{A}{\mu_{0}},\;P\left(t\right)\leq k-cG_{m},\;G\left(t\right)\leq \frac{Q}{\mu_{1}}\right\}.$$

### Equilibria analysis

This section identifies and analyzes the potential equilibrium points and their stability. To accomplish this, we compute $\frac{dD}{dt}=\frac{dP}{dt}=\frac{dG}{dt}=0$ and obtain the following equilibrium

1 The desertification point $Z_{1}=\left(D_{1},\;0,G_{1}\right)$, where $D_{1}=\frac{A}{\mu_{0}}$ and $G_{1}=\frac{Q\mu_{0}}{A\gamma_{1}+\mu_{0}\mu_{1}}$.
2 The non-desertification point $Z_{2}=\left(D_{2},\;P_{2},\;G_{2}\right)$, where $D_{2}=\frac{A}{\mu_{0}+\alpha P},\;G_{2}=\frac{Q(\mu_{0}+\alpha P)}{\alpha \gamma_{2}P^{2}+\left(\mu_{0}\gamma_{2}+\mu_{1}\alpha \right)P+\left(A\gamma_{1}+\mu_{0}\mu_{1}\right)}\;$and $P_{2}\;$is the root of f(P), where f(P) is
  $$f\left(P\right)=e_{1}P^{4}+e_{2}P^{3}+e_{3}P^{2}+e_{4}P+e_{5}=0,$$

here,

$$e_{1}=-r\alpha^{2}\gamma_{2}$$

$$e_{2}=r\alpha \left(k\alpha \gamma_{2}-2\mu_{0}\gamma_{2}-\mu_{1}\alpha \right)$$

$$e_{3}=k\alpha \gamma_{2}\left(r\mu_{0}-\beta A\right)+r\left(\mu_{0}\gamma_{2}+\mu_{1}\alpha \right)\left(k\alpha -\mu_{0}\right)-r\alpha \left(A\gamma_{1}+\mu_{0}\mu_{1}+cQ\alpha \right)$$

$$e_{4}=k\left(\mu_{0}\gamma_{2}+\mu_{1}\alpha \right)\left(r\mu_{0}-\beta A\right)-r\left(A\gamma_{1}+\mu_{0}\mu_{1}\right)\left(k\alpha -\mu_{0}\right)-cQ\alpha \left(2r\mu_{0}-\beta A\right)$$

$$e_{5}=\left(r\mu_{0}-\beta A\right)\left(k\left(A\gamma_{1}+\mu_{0}\mu_{1}\right)-cQ\mu_{0}\right)$$

Clearly, $f\left(0\right)=\left(r\mu_{0}-\beta A\right)\left(k\left(A\gamma_{1}+\mu_{0}\mu_{1}\right)-cQ\mu_{0}\right)$, and $f\left(k\right)=e_{1}k^{4}+e_{2}k^{3}+e_{3}k^{2}+e_{4}k+e_{5}$*.*

So, f(P) has a unique positive root, say $P_{2}$, where $P_{2}\in \left(0,\;k\right)\;$if one of the following cases is satisfied

(2) $$\begin{array}{c} f\left(0\right)>0,\;f\left(k\right)<0\;\text{and}\;f^{\prime }\left(P\right)<0,\\ f\left(0\right)\langle 0,\;f\left(k\right)\rangle 0\;\text{and}\;f^{\prime }\left(P\right)>0. \end{array}$$

### Stability analysis of the DPG model

To assess the linear stability of the DPG system, it is essential to compute the Jacobian matrix, which is defined as

(3) $$J=\left[\begin{array}{ccc} -\mu_{0}-\alpha P & -\alpha D & 0\\ -\beta P & r-\frac{2rP}{k-cG}-\beta D & -\frac{rcP^{2}}{{\left(k-cG\right)}^{2}}\\ -\gamma_{1}G & -\gamma_{2}G & -\left(\gamma_{1}D+\gamma_{2}P+\mu_{1}\right) \end{array}\right].$$

Around the two equilibrium points indicated above, the local analysis of the DPC model is figured out as

$J\left(Z_{1}\right)=J\left(D_{1},0,G_{1}\right)$ is given as:

(4) $$J\left(Z_{1}\right)=\left[\begin{array}{ccc} -\mu_{0} & -\frac{\alpha A}{\mu_{0}} & 0\\ 0 & r-\frac{\beta A}{\mu_{0}} & 0\\ \frac{-\mu_{0}\gamma_{1}Q}{\gamma_{1}A+\mu_{0}\mu_{1}} & \frac{-\mu_{0}\gamma_{2}Q}{\gamma_{1}A+\mu_{0}\mu_{1}} & -\frac{\gamma_{1}A}{\mu_{0}}-\mu_{1} \end{array}\right],$$

The characteristic equation of $J(Z_{1})$ is $\left(-\mu_{0}-\lambda \right)\left(r-\frac{\beta A}{\mu_{0}}-\lambda \right)\left(-\frac{\gamma_{1}A}{\mu_{0}}-\mu_{1}-\lambda \right)$, and the eigenvalues of $J(Z_{1})$ are $\lambda_{1}=-\mu_{0}<0$, $\lambda_{2}=r-\frac{\beta A}{\mu_{0}}$ and $\lambda_{3}=-\frac{\gamma_{1}A}{\mu_{0}}-\mu_{1}<0$. Therefore, $Z_{1}$ is asymptotic stable if

(5) $$r<r^{*},$$

where, $r^{*}=\frac{\beta A}{\mu_{0}}$. Condition 5 shows that desertification could happen when the intrinsic growth rate of plant biomass is less than the plant biomass depletion rate coefficient due to dust pollutants. Conversely, for $r>\frac{\beta A}{\mu_{0}}$, $Z_{1}$ is a saddle point. For $r=\frac{\beta A}{\mu_{0}}$, then $J(Z_{1})$ has zero eigenvalue, creation $Z_{1}$ a nonhyperbolic point.

3. $\;J\left(Z_{2}\right)=J\left(D_{2},P_{2},G_{2}\right)\;$is given as:
  (6) $$J\left(Z_{2}\right)=\left(\begin{array}{ccc} -\left(\mu_{0}+\alpha P_{2}\right) & -\alpha D_{2} & 0\\ -\beta P_{2} & -\frac{rP_{2}}{\left(k-cG_{2}\right)} & -\frac{rcP_{2}^{2}}{{\left(k-cG_{2}\right)}^{2}}\\ -\gamma_{1}G_{2} & -\gamma_{2}G_{2} & -\left(\gamma_{1}D_{2}+\gamma_{2}P_{2}+\mu_{1}\right) \end{array}\right).$$

So, the eigenvalues of $\left(Z_{2}\right)$ are the roots of the following equation

(7) $$\left(\lambda^{3}+S_{1}\lambda^{2}+S_{2}\lambda +S_{3}\right)=0$$

where:

$$S_{1}=-\left(z_{11}+z_{22}+z_{33}\right)=\mu_{0}+\mu_{1}+\gamma_{1}D_{2}+\left(\alpha +\gamma_{2}+\frac{r}{\left(k-cG_{2}\right)}\right)p_{2}>0,$$

$$\begin{array}{ccc} S_{2} & = & z_{11}\left(z_{22}+z_{33}\right)+z_{22}z_{33}-z_{23}z_{32}-z_{12}z_{21}=\left(\mu_{0}+\alpha p_{2}\right)\left(\frac{rP_{2}}{\left(k-cG_{2}\right)}+\gamma_{1}D_{2}+\gamma_{2}P_{2}+\mu_{1}\right)\\ & & \;+\left(\frac{rP_{2}}{\left(k-cG_{2}\right)}\right)\left(\gamma_{1}D_{2}+\gamma_{2}P_{2}+\mu_{1}\right)-\gamma_{2}G_{2}\left(\frac{rcP_{2}^{2}}{{\left(k-cG_{2}\right)}^{2}}\right)-\alpha \beta D_{2}P_{2}, \end{array}$$

$$S_{3}=z_{11}\left(z_{23}z_{32}-z_{22}z_{33}\right)+z_{12}\left(z_{21}z_{33}-z_{23}z_{31}\right)$$

$$=-\left(\mu_{0}+\alpha p_{2}\right)\left[\left(\frac{rc\gamma_{2}G_{2}P_{2}^{2}}{{\left(k-cG_{2}\right)}^{2}}\right)-\frac{\left(\gamma_{1}D_{2}+\gamma_{2}P_{2}+\mu_{1}\right)rP_{2}}{\left(k-cG_{2}\right)}\right]-\alpha D_{2}\left[\beta P_{2}\left(\gamma_{1}D_{2}+\gamma_{2}P_{2}+\mu_{1}\right)-\frac{rc\gamma_{1}G_{2}P_{2}^{2}}{{\left(k-cG_{2}\right)}^{2}}\right],$$

$$S_{1}S_{2}-S_{3}=\left(z_{11}+z_{22}\right)\left(z_{12}z_{21}-z_{33}^{2}\right)+\left(z_{22+}z_{33}\right)\left(z_{23}z_{32}-z_{11}^{2}\right)$$

$$-z_{22}^{2}\left(z_{11}+z_{33}\right)-2z_{11}z_{22}z_{33}+z_{12}z_{23}z_{31}$$

$$\begin{array}{ccc} & = & -\left(\mu_{0}+\alpha P_{2}+\frac{rP_{2}}{\left(k-cG_{2}\right)}\right)\left(\alpha \beta P_{2}D_{2}-{\left(\gamma_{1}D_{2}+\gamma_{2}P_{2}+\mu_{1}\right)}^{2}\right)-\left(\gamma_{1}D_{2}+\gamma_{2}P_{2}+\mu_{1}+\frac{rP_{2}}{\left(k-cG_{2}\right)}\right)\left(\frac{rc\gamma_{2}G_{2}P_{2}^{2}}{{\left(k-cG_{2}\right)}^{2}}-{\left(\mu_{0}+\alpha P_{2}\right)}^{2}\right)\\ & & \;+{\left(\frac{rP_{2}}{\left(k-cG_{2}\right)}\right)}^{2}\left(\mu_{0}+\alpha P_{2}+\gamma_{1}D_{2}+\gamma_{2}P_{2}+\mu_{1}\right)+2\left(\left(\mu_{0}+\alpha P_{2}\right)\left(\frac{rP_{2}}{\left(k-cG_{2}\right)}\right)\left(\gamma_{1}D_{2}+\gamma_{2}P_{2}+\mu_{1}\right)\right)-\frac{rc\alpha \gamma_{1}G_{2}D_{2}P_{2}^{2}}{{\left(k-cG_{2}\right)}^{2}} \end{array}$$

Thus, by the Routh-Hurwitz rule [34], $Z_{2}$ is asymptotically stable if $S_{3}>0$ and $S_{1}S_{2}>S_{3}.$

### Global stability

This section will analyze global stability (GAS) around equilibrium points to investigate the dynamics of the DPC model in regions distant from these points using the Lyapunov direct method [35].

Theorem 3 $\;Z_{1}=\left(D_{1},0,G_{1}\right)$ *is a GAS provided the following conditions hold:*

(8) $$\begin{array}{c} \begin{array}{c} \frac{\left(\gamma_{1}D+\mu_{1}\right)}{4}\geq max\left\{\frac{{\left(\gamma_{1}G_{1}\right)}^{2}}{\mu_{0}},\frac{{\gamma_{2}}^{2}G^{2}\left(k-cG\right)}{r}\right\} \end{array}\\ \;\\ \frac{4\left(k-cG\right){\left(\alpha D\right)}^{2}}{\mu_{0}}\leq r<\beta D\; \end{array}\}$$

Proof: Let us define a Lyapunov function for the DPG model around $Z_{1}$ as:

$$L_{1}\left(t\right)=\frac{{\left(D-D_{1}\right)}^{2}}{2}+P+\frac{{\left(G-G_{1}\right)}^{2}}{2}$$

where *L*_1_(*t*) is a positive definite about *Z*_1_. Thus,

$$\begin{array}{ccc} \frac{dL_{1}}{dt} & = & \left(D-D_{1}\right)\frac{dD}{dt}+\frac{dP}{dt}+\left(G-G_{1}\right)\frac{dG}{dt}=\left(D-D_{1}\right)\left(A-\mu_{0}D-\alpha DP-A+\mu_{0}D\right)+\left(rP-\frac{rP^{2}}{k-cG}-\beta DP\right)\\ & & \;+\left(G-G_{1}\right)\left(Q-\gamma_{1}DG-\gamma_{2}PG-\mu_{1}G-Q+\gamma_{1}D_{1}G_{1}+\mu_{1}G_{1}\right). \end{array}$$

Therefore,

$$\frac{dL_{1}}{dt}=\left(D-D_{1}\right)\left(-\mu_{0}\left(D-D_{1}\right)-\alpha DP\right)+\left(rP-\frac{rP^{2}}{k-cG}-\beta DP\right)+\left(G-G_{1}\right)\left(-\gamma_{1}\left(DG-D_{1}G_{1}\right)-\gamma_{2}PG-\mu_{1}\left(G-G_{1}\right)\right).$$

i.e.,

$$\frac{dL_{1}}{dt}=-\mu_{0}{\left(D-D_{1}\right)}^{2}-\alpha DP\left(D-D_{1}\right)+rp-\frac{rP^{2}}{k-cG}-\beta DP-\left(\gamma_{1}D+\mu_{1}\right){\left(G-G_{1}\right)}^{2}-\gamma_{1}G_{1}\left(D-D_{1}\right)\left(G-G_{1}\right)-\gamma_{2}PG\left(G-G_{1}\right).$$

$$\begin{array}{ccc} & & \Rightarrow \frac{dL_{1}}{dt}=-[\frac{\mu_{0}{\left(D-D_{1}\right)}^{2}}{2}+\alpha DP\left(D-D_{1}\right)+\frac{r}{2\left(k-cG\right)}P^{2}+\frac{r}{2\left(k-cG\right)}P^{2}+\gamma_{2}PG\left(G-G_{1}\right)+\frac{\left(\gamma_{1}D+\mu_{1}\right)}{2}{\left(G-G_{1}\right)}^{2}\\ & & +\frac{\left(\gamma_{1}D+\mu_{1}\right)}{2}{\left(G-G_{1}\right)}^{2}+\gamma_{1}G_{1}\left(D-D_{1}\right)\left(G-G_{1}\right)+\frac{\mu_{0}{\left(D-D_{1}\right)}^{2}}{2}-P\left(r-\beta D\right)]. \end{array}$$

Consequently,

$$\frac{dL_{1}}{dt}=-{\left(\sqrt{\frac{\mu_{0}}{2}}\left(D-D_{1}\right)+\sqrt{\frac{r}{2\left(k-cG\right)}}\;P\right)}^{2}-{\left(\sqrt{\frac{r}{2\left(k-cG\right)}\;}P+\sqrt{\frac{\left(\gamma_{1}D-\mu_{1}\right)}{2}}\left(G-G_{1}\right)\right)}^{2}-{\left(\sqrt{\frac{\left(\gamma_{1}D-\mu_{0}\right)}{2}}\left(G-G_{1}\right)+\sqrt{\frac{\mu_{0}}{2}}\left(D-D_{1}\right)\right)}^{2}.$$

So, $dL_{1}/dt<0$ under condition (8) and hence $L_{1}\left(t\right)$ is a Lyapunov function. Thus, $\;Z_{1}=\left(D_{1},0,G_{1}\right)$ is GAS in $R_{+}^{3}$ if D and G are controlled as in condition (8).

Consequently, the desertification point satisfies the criteria for local stability, hence establishing its global stability. From a biological standpoint, the increase in dust pollutants and global warming may lead to the eradication of green spaces and the transformation into desertified regions if the specific criteria in (8) are satisfied.

Theorem 4 $Z_{2}=\left(D_{2},P_{2},G_{2}\right)$ *is a GAS provided the following conditions hold:*

(9) $$\begin{array}{c} \frac{\left(\mu_{0}+\alpha P_{2}\right)}{4}\geq Max\left[\frac{{\left(\alpha D+\beta \right)}^{2}\left(k-cG\right)}{r},\frac{\gamma_{1}^{2}G_{2}^{2}}{\gamma_{1}D+\gamma_{2}P+\mu_{1}}\right]\\ {\left(\frac{rcP_{2}}{\left(k-cG\right)\left(k-cG_{2}\right)}+\gamma_{2}G_{2}\right)}^{2}\leq \frac{r\left(\gamma_{1}D+\gamma_{2}P+\mu_{2}\right)}{k-cG} \end{array}\}.$$

**Proof**: Let us define a Lyapunov function for the DPG model around $Z_{2}$ as:

$$L_{2}=\frac{{\left(D-D_{2}\right)}^{2}}{2}+\left(P-P_{2}-P_{2}\ln\frac{P}{P_{2}}\right)+\frac{{\left(G-G_{2}\right)}^{2}}{2},$$

where $L_{2}\left(t\right)$ is a positive definite about $Z_{2}$. Thus,

$$\frac{dL_{2}}{dt}=\left(D-D_{2}\right)\frac{dD}{dt}+\left(\frac{P-P_{2}}{P}\right)\frac{dP}{dt}+\left(G-G_{2}\right)\frac{dG}{dt},$$

Therefore,

$$\begin{array}{ccc} & & \frac{dL_{2}}{dt}=\left(D-D_{2}\right)\left[A-\mu_{0}D-\alpha DP-A+\mu_{0}D_{2}+\alpha D_{2}P_{2}\right]+\left(P-P_{2}\right)\left[r-\frac{rp}{k-cG}-\beta D-r+\frac{rP_{2}}{k-cG_{2}}+\beta D_{2}\right]\\ & & +\left(G-G_{2}\right)\left[Q-\gamma_{1}DG-\gamma_{2}PG-\mu_{1}G-Q+\gamma_{1}D_{2}G_{2}+\gamma_{2}P_{2}G_{2}+\mu_{1}G_{2}\right]. \end{array}$$

Thus,

$$\begin{array}{ccc} \left(dL\_2\right)/dt & = & -\left(\mu \_0+\alpha P\_2\;\right)\;{\left(D-D\_1\;\right)}^{2}-\alpha D\left(P-P\_2\;\right)\left(D-D\_2\;\right)-\left(r{\left(P-P\_2\;\right)}^{2}\right)/\left(k-cG\right)-\left(rcP\_2\;\left(G-G\_2\;\right)\left(P-P\_2\;\right)\right)/\\ & & \left(k-cG\right)\left(k-cG\_2\;\right)\;-\beta \left(D-D\_2\;\right)\left(P-P\_2\;\right)-\left(\gamma \_1\;D+\gamma \_2\;P+\mu \_1\;\right)\;{\left(G-G\_1\;\right)}^{2}-\gamma \_1\;G\_2\;\left(D-D\_2\;\right)\left(G-G\_2\;\right)\\ & & -\gamma \_2\;G\_2\;\left(P-P\_2\;\right)\left(G-G\_2\;\right). \end{array}$$

i.e.,

$$\begin{array}{ccc} \frac{dL_{2}}{dt} & = & -[\frac{\left(\mu_{0}+\alpha P_{2}\right)}{2}{\left(D-D_{2}\right)}^{2}+\left(\alpha D+\beta \right)\left(D-D_{2}\right)\left(P-P_{2}\right)+\frac{r}{2\left(k-cG\right)}{\left(P-P_{2}\right)}^{2}\\ & & \;+\frac{r}{2\left(k-cG\right)}{\left(P-P_{2}\right)}^{2}+\left(\frac{rcP_{2}}{\left(k-cG\right)\left(k-cG_{2}\right)}+\gamma_{2}G_{2}\right)\left(P-P_{2}\right)\left(G-G_{2}\right)+\frac{\left(\gamma_{1}D+\gamma_{2}P_{2}+\mu_{1}\right)}{2}{\left(G-G_{2}\right)}^{2}\\ & & \;+\frac{\left(\gamma_{1}D+\gamma_{2}P_{2}+\mu_{1}\right)}{2}{\left(G-G_{2}\right)}^{2}+\gamma_{1}G_{2}\left(D-D_{2}\right)\left(G-G_{2}\right)+\frac{\left(\mu_{0}+\alpha P_{2}\right)}{2}{\left(D-D_{2}\right)}^{2}]. \end{array}$$

Therefore,

$$\begin{array}{ccc} & & \frac{dL_{2}}{dt}\leq -{\left[\sqrt{\frac{\left(\mu_{0}+\alpha P_{2}\right)}{2}}\left(D-D_{2}\right)+\sqrt{\frac{r}{2\left(k-cG\right)}}\left(P-P_{2}\right)\right]}^{2}-{\left[\sqrt{\frac{r}{2\left(k-cG\right)}}\left(P-P_{2}\right)+\sqrt{\frac{(\gamma_{1}D+\gamma_{2}P+\mu_{1}}{2}}\left(G-G_{2}\right)\right]}^{2}\\ & & \;-{\left[\sqrt{\frac{(\gamma_{1}D+\gamma_{2}P+\mu_{1}}{2}}\left(G-G_{2}\right)+\sqrt{\frac{\left(\mu_{0}+\alpha P_{2}\right)}{2}}\left(D-D_{2}\right)\right]}^{2} \end{array}$$

Thus, $dL_{2}/dt<0$ under condition (9) and hence $L_{2}\left(t\right)$ is a Lyapunov function. Consequently, $Z_{2}=\left(D_{2},P_{2},G_{2}\right)$ is GAS in $R_{+}^{3}$ if D,P and G are controlled as in condition (9).

From a biological perspective, condition (9) ensures that green spaces persist despite the increasing levels of dust pollutants and global warming.

### Local bifurcation

This section examines the local bifurcation around the steady states utilizing Sotomayor's rule for local bifurcation; for instance, see [19,[36], [37], [38]].

Theorem 5 *For*$r^{*}=\frac{\beta A}{\mu_{0}}$*, the DPG model, at*$Z_{1}$*has a transcritical bifurcation (*TB*)*.

**Proof.** At $r^{*}=\frac{\beta A}{\mu_{0}}$, $J(Z_{1})$ has a zero eigenvalue $\lambda_{2}^{1}=0$. So, $J(Z_{1})$ at $r^{*}$ becomes

$$J^{*}\left(Z_{1}\right)=-\left[\begin{array}{ccc} \mu_{0} & \frac{A\alpha }{\mu_{0}} & 0\\ 0 & 0 & 0\\ \frac{Q\mu_{0}\gamma_{1}}{A\gamma_{1}+\mu_{0}\mu_{1}} & \frac{Q\mu_{0}\gamma_{2}}{A\gamma_{1}+\mu_{0}\mu_{1}} & \frac{\left(A\gamma_{1}+\mu_{0}\mu_{1}\right)}{\mu_{0}} \end{array}\right]$$

Now, let $W^{\left[1\right]}={\left(w_{1}^{\left[1\right]},w_{2}^{\left[2\right]},w_{3}^{\left[3\right]}\right)}^{T}$ and $Y^{\left[1\right]}={\left(y_{1}^{\left[1\right]},y_{2}^{\left[2\right]},y_{3}^{\left[1\right]}\right)}^{T}$ are the eigenvectors corresponding to $\lambda_{2}^{1}=0$ of $J^{*}\left(Z_{1}\right)$ and ${J^{*}}^{T}\left(Z_{1}\right)$ respectively. The computations give $W^{\left[1\right]}={\left(\frac{-A\alpha }{\mu_{0}^{2}},1,\frac{-Q(A\alpha \gamma_{1}-\gamma_{2}\mu_{0}^{2})}{{\left(A\gamma_{1}+\mu_{0}\mu_{1}\right)}^{2}}\;\right)}^{T}$ and $Y^{\left[0\right]}={\left(\;0,1,0\right)}^{T}$.

Now, let $f={\left(f_{1}\left(D,P\right),f_{2}\left(D,P,G\right),f_{3}\left(D,P,G\right)\right)}^{T}$, then

$$\frac{\partial f}{\partial r}=\left(\frac{\partial f_{1}}{\partial r},\;\frac{\partial f_{2}}{\partial r},\;\frac{\partial f_{3}}{\partial r}\right)=\left(0,P-\frac{P^{2}}{k-cG},0\right)\Rightarrow \;f_{r}\left(z_{1},\;r^{*}\right)=\left(0,0,0\right).$$

Hence,

$$\;{Y^{\left[1\right]}}^{T}f_{r}\left(Z_{1},r^{*}\right)=\left(0,1,0\right){\left(0,0,0\right)}^{T}=0.$$

That means the (SNB) cannot happen at $r^{*}$, while the first condition of TB is satisfied.

Subsequently,

$$\;{Y^{\left[1\right]}}^{T}\left[Df_{r}\left(Z_{1},r^{*}\right)w^{\left[1\right]}\right]=\left(0,1,0\right)\left[\begin{array}{ccc} 0 & 0 & 0\\ 0 & 1 & 0\\ 0 & 0 & 0 \end{array}\right]\;\left(\begin{array}{c} \frac{-A\alpha }{\mu_{0}^{2}}\\ 1\\ \frac{Q\left(A\alpha \gamma_{1}-\gamma_{2}\mu_{0}^{2}\right)}{\left(A\gamma_{1}+\mu_{0}\mu_{1}\right){\;}^{2}} \end{array}\right)=1\neq 0,$$

$$\;{Y^{\left[1\right]}}^{T}\left[D^{2}f_{r}\left(Z_{1},r^{*}\right)\left(w^{\left[1\right]},w^{\left[1\right]}\right)\right]=\left(0,1,0\right)\left[-2{\left(\alpha w_{1}^{\left[1\right]}w_{2}^{\left[1\right]},w_{2}^{\left[1\right]}\left(\beta w_{1}^{\left[1\right]}+\frac{r^{*}\;w_{2}^{\left[1\right]}}{k-cG_{1}}\right),w_{3}^{\left[1\right]}\left(\gamma_{1}w_{1}^{\left[1\right]}+\gamma_{2}w_{2}^{\left[1\right]}\right)\right)}^{T}\right]=-2r^{*}\left(\frac{\alpha }{\mu_{0}}+\frac{1}{k-cG_{1}}\;\right)\neq 0$$

According to Sotomayor's theorem, the DPG faces TB at $Z_{1}$ for $r^{*}$.

Theorem 6 *For*$\alpha^{*}=\frac{\left(z_{22}^{\left[2\right]}+z_{33}^{\left[2\right]}\right)\left(z_{23}^{\left[2\right]}z_{32}^{\left[2\right]}-{\left[z_{11}^{\left[2\right]}\right]}^{2}\;\right)-{\left[z_{33}^{\left[2\right]}\right]}^{2}\left(z_{11}^{\left[2\right]}+z_{22}^{\left[2\right]}\right)-{\left[z_{22}^{\left[2\right]}\right]}^{2}\left(z_{11}^{\left[2\right]}+z_{33}^{\left[2\right]}\right)-2z_{11}^{\left[2\right]}z_{22}^{\left[2\right]}z_{33}^{\left[2\right]}}{D_{2}\left(z_{11}^{\left[2\right]}z_{21}^{\left[2\right]}+z_{22}^{\left[2\right]}z_{21}^{\left[2\right]}+z_{23}^{\left[2\right]}z_{31}^{\left[2\right]}\right)}$*, where*$\alpha^{*}>0$*, the DPG model, at*$Z_{2}$*has SNB if*

(10) $${\left(Y^{\left[2\right]}\right)}^{T}\left[D^{2}f_{\alpha }\left(Z_{2},\alpha^{*}\right)\left(w^{\left[2\right]},w^{\left[2\right]}\right)\right]\neq 0,$$

where the notation in (10) will be measured in the following proof and the formulas of $z_{ij}^{\left[2\right]}$ are given in (6).

Proof According to $J(Z_{2})$, it is observed that $S_{1}S_{2}-S_{3}=0$ gives $\alpha^{*}$ and $J^{*}\left(Z_{2}\right)=J\left(Z_{2},\alpha^{*}\right)$, becomes**:**

$$J^{*}\left(Z_{2}\right)=\left[\begin{array}{ccc} \phi_{11} & \phi_{12} & 0\\ \phi_{21} & \phi_{22} & \phi_{23}\\ \phi_{31} & \phi_{32} & \phi_{33} \end{array}\;\right]$$

here,

$$\phi_{11}=-\left(\mu_{0}+\alpha^{*}P_{2}\right),\phi_{12}=-\alpha^{*}D_{2},$$

$$\phi_{21}=-\beta^{*}P_{2},\phi_{22}=-\frac{rP_{2}}{\left(k-cG_{2}\right)},\phi_{23}=-\frac{rcP_{2}^{2}}{{\left(k-cG_{2}\right)}^{2}},$$

$$\phi_{31}=-\gamma_{1}G_{2},\;\phi_{32}=-\gamma_{2}G_{2},\;\phi_{33}=-\left(\gamma_{1}D_{2}+\gamma_{2}P_{2}+\mu_{1}\right).$$

Now, let $W^{\left[2\right]}={\left(w_{1}^{\left[2\right]},w_{2}^{\left[2\right]},w_{3}^{\left[2\right]}\right)}^{T}$ and $Y^{\left[2\right]}={\left(y_{1}^{\left[2\right]},y_{2}^{\left[2\right]},y_{3}^{\left[2\right]}\right)}^{T}$ represent the eigenvectors corresponding to $\lambda_{1}^{3}=0$ of $J^{*}\left(Z_{2}\right)$ and ${J^{*}}^{T}\left(Z_{2}\right)$ respectively. The computations give $W^{\left[2\right]}={\left(\frac{-\phi_{12}}{\phi_{11}},1,\frac{\phi_{11}\phi_{22}-\phi_{12}\phi_{21}}{\phi_{11}\phi_{33}}\right)}^{T}$ and $Y^{\left[2\right]}={\left(\frac{\phi_{23}\phi_{31}-\phi_{21}\phi_{33}}{\phi_{11}\phi_{33}},1,\frac{-\phi_{23}}{\phi_{33}}\right)}^{T}$.

Subsequently,

$$\;{Y^{\left[2\right]}}^{T}f_{\alpha }\left(Z_{2},\alpha^{*}\right)=\left(\frac{\phi_{23}\phi_{31}-\phi_{21}\phi_{33}}{\phi_{11}\phi_{33}},1,\frac{-\phi_{23}}{\phi_{33}}\right){\left(-D_{2}P_{2},0,0\right)}^{T}=\frac{\phi_{23}\phi_{31}-\phi_{21}\phi_{33}}{\phi_{11}\phi_{33}}D_{2}P_{2}\neq 0,$$

$$\begin{array}{ccc} & & {\left(Y^{\left[2\right]}\right)}^{T}\left[D^{2}f_{\alpha }\left(Z_{2},\alpha^{*}\right)\left(w^{\left[2\right]},w^{\left[2\right]}\right)\right]=\left(\frac{\phi_{23}\phi_{31}-\phi_{21}\phi_{33}}{\phi_{11}\phi_{33}},1,\frac{-\phi_{23}}{\phi_{33}}\right)\\ & & \;-2{\left(\alpha^{*}w_{1}^{\left[2\right]}w_{2}^{\left[2\right]},w_{2}^{\left[2\right]}\left(\beta w_{1}^{\left[2\right]}+\frac{rw_{2}^{\left[2\right]}}{k-cG_{2}}\right)+\frac{rcPw_{3}^{\left[2\right]}\left(2\left(k-cG_{2}\right)+cPw_{3}^{\left[2\right]}\right)}{{\left(k-cG_{2}\right)}^{3}},w_{3}^{\left[2\right]}\left(\gamma_{1}w_{1}^{\left[2\right]}+\gamma_{2}w_{2}^{\left[2\right]}\right)\right)}^{T} \end{array}$$

$$=-2\left(\alpha^{*}w_{1}^{\left[2\right]}\left(\frac{\phi_{23}\phi_{31}-\phi_{21}\phi_{33}}{\phi_{11}\phi_{33}}\right)+\left[\beta w_{1}^{\left[2\right]}+\frac{r}{k-cG_{2}}+\frac{rcP_{2}w_{3}^{\left[2\right]}\left(2\left(k-cG_{2}\right)+cpw_{3}^{\left[2\right]}\right)}{{\left(k-cG_{2}\right)}^{3}}\right]+w_{3}^{\left[2\right]}\left(\gamma_{1}w_{1}^{\left[2\right]}+\gamma_{2}\right)\left(\frac{-\phi_{23}}{\phi_{33}}\right)\right).$$

Therefore, condition (10) guarantees that the SNB is taken place at $Z_{2}$ with the parameter $\alpha^{*}$.

Theorem 7 *Under the following assumptions*

(11) $$S_{i}>0,\;i=1,2$$

(12) $$\gamma_{1}^{*}>0$$

where $S_{i},\;i=1,2$ are specified in Eq. (7) with $\gamma_{1}=\gamma_{1}^{*}$ and the formulation of $\gamma_{1}^{*}$ is given in the following proof. Then, the DPG system undergoes a Hopf bifurcation (HB) for $Z_{2}$ at $\gamma_{1}=\gamma_{1}^{*}$.

Proof: To find the bifurcation parameter $\gamma_{1}^{*}$, we set $S_{1}\left(\gamma_{1}^{*}\right)S_{2}\left(\gamma_{1}^{*}\right)-S_{3}\left(\gamma_{1}^{*}\right)=0$. This gives:

$$\gamma_{1}^{*}=\frac{2z_{11}z_{22}z_{33}+z_{22}^{2}\left(z_{11}+z_{33}\right)-\left(z_{22}+z_{33}\right)\left(z_{23}z_{32}-z_{11}^{2}\right)-\left(z_{11}+z_{22}\right)\left(z_{12}z_{21}-z_{33}^{2}\right)}{G_{2}z_{12}z_{23}}.$$

Clearly, $\gamma_{1}^{*}>0$ if condition (12) holds. At $\gamma_{1}=\gamma_{1}^{*}$, Eq. (7) can be written as

$$\left(\lambda +S_{1}\right)\left(\lambda^{2}+S_{2}\right)=0.$$

The above equation has the following roots: a negative root $\lambda_{1}=-S_{1}$ and two purely imaginary roots $\;\lambda_{2,3}=\pm i\sqrt{S_{2}}$ if condition (11) is satisfied. In a neighbourhood of $\gamma_{1}^{*}$, the roots have the following forms: $\lambda_{1}=-S_{1},\;\lambda_{2,3}=\chi_{1}\left(\gamma_{1}\right)\pm i\chi_{2}\left(\gamma_{1}\right)$.

The following are calculated to denote the conditions for HB to occur at $\gamma_{1}=\gamma_{1}^{*}$:

1. $Re\left(\;\lambda_{2,3}\right){|}_{\gamma_{1}=\gamma_{1}^{*}}=\chi_{1}\left(\gamma_{1}^{*}\right)=0$
2. To calculate the transversality condition, $\Theta \left(\gamma_{1}^{*}\right)\psi \left(\gamma_{1}^{*}\right)+\Gamma \left(\gamma_{1}^{*}\right)\phi \left(\gamma_{1}^{*}\right)\neq 0$, we substitute $\chi_{1}\left(\gamma_{1}\right)\pm i\chi_{2}\left(\gamma_{1}\right)$ into Eq. (7), where the form of $\Theta \left(\gamma_{1}\right),\;\psi \left(\gamma_{1}\right),\;\Gamma \left(\gamma_{1}\right)$ and $\phi (\gamma_{1})$ are
  $$\psi \left(\gamma_{1}\right)=3\chi_{1}^{2}\left(\gamma_{1}\right)+2S_{1}\left(\gamma_{1}\right)\chi_{1}\left(\gamma_{1}\right)+S_{2}\left(\gamma_{1}\right)-3\chi_{2}^{2}\left(\gamma_{1}\right),$$
  $$\phi \left(\gamma_{1}\right)=6\chi_{1}\left(\gamma_{1}\right)\chi_{2}\left(\gamma_{1}\right)+2S_{1}\left(\gamma_{1}\right)\chi_{2}\left(\gamma_{1}\right),$$
  $$\Theta \left(\gamma_{1}\right)=\chi_{1}^{2}\left(\gamma_{1}\right)S_{1}{\;}^{\prime }\left(\gamma_{1}\right)+S_{2}{\;}^{\prime }\left(\gamma_{1}\right)\chi_{1}\left(\gamma_{1}\right)+S_{3}{\;}^{\prime }\left(\gamma_{1}\right)-S_{1}{\;}^{\prime }\left(\gamma_{1}\right)\chi_{2}^{2}\left(\gamma_{1}\right),$$
  $$\Gamma \left(\gamma_{1}\right)=2\chi_{1}\left(\gamma_{1}\right)\chi_{2}\left(\gamma_{1}\right)S_{1}^{\prime }\left(\gamma_{1}\right)+S_{2}^{\prime }\left(\gamma_{1}\right)\chi_{2}\left(\gamma_{1}\right).$$

Now at $\gamma_{1}=\gamma_{1}^{*}$, substitution $\chi_{1}=0$ and $\chi_{2}=\sqrt{S_{2}}$, the following is obtained:

$$\begin{array}{cc} & \psi \left(\gamma_{1}^{*}\right)=-2S_{2}\left(\gamma_{1}^{*}\right),\\ & \phi \left(\gamma_{1}^{*}\right)=2S_{1}\left(\gamma_{1}^{*}\right)\sqrt{S_{2}\left(\gamma_{1}^{*}\right)},\\ & \Theta \left(\gamma_{1}^{*}\right)=S_{3}^{\prime }\left(\gamma_{1}^{*}\right)-S_{1}^{\prime }\left(\gamma_{1}^{*}\right)S_{2}\left(\gamma_{1}^{*}\right),\\ & \Gamma \left(\gamma_{1}^{*}\right)=S_{2}^{\prime }\left(\gamma_{1}^{*}\right)\sqrt{S_{2}\left(\gamma_{1}^{*}\right)}, \end{array}$$

where,

$$S_{1}^{\prime }\left(\gamma_{1}^{*}\right)=0,$$

$$S_{2}^{\prime }\left(\gamma_{1}^{*}\right)=0,$$

$$S_{3}^{\prime }\left(\gamma_{1}^{*}\right)=G_{2}z_{12}z_{23}.$$

Hence,

$$\Theta \left(\gamma_{1}^{*}\right)\psi \left(\gamma_{1}^{*}\right)+\Gamma \left(\gamma_{1}^{*}\right)\phi \left(\gamma_{1}^{*}\right)=-2G_{2}z_{12}z_{23}S_{2}\left(\gamma_{1}^{*}\right)\neq 0.$$

So, the HB has occurred at $\gamma_{1}^{*}$.

The stability condition of the stable limit cycle in $R_{\left(D,P,G\right)}^{3}$ is presented in the following theorem by using the coefficient of curvature of the limit cycle [39].

Theorem 8 *The DPG model has a stable limit cycle in* $R_{\left(D,P,G\right)}^{3}$ *if the following condition is true:*

(13) $$k-c\left(x_{3}+G_{2}\right)>0.$$

Proof: by shifting $Z_{2}=\left(D_{2},P_{2},G_{2}\right)$ to (0,0,0) by using the following transformations $D=x_{1}+D_{2}$, $P=x_{2}+P_{2}$, $G=x_{3}+D_{2}$. Then, the DPG system becomes:

$$\frac{dx_{1}}{dt}=-\alpha x_{1}x_{2}$$

$$\frac{dx_{2}}{dt}=-\frac{r{\left(x_{2}+P_{2}\right)}^{2}}{k-c\left(x_{3}+G_{2}\right)}-\beta \left(x_{1}x_{2}\right)$$

$$\frac{dx_{3}}{dt}=-\gamma_{1}x_{1}x_{3}-\gamma_{2}x_{2}x_{3}.$$

The following matrix offers the nonlinear part of the above system:

$$\eta =\left(\begin{array}{c} \eta_{1}\\ \eta_{2}\\ \eta_{3} \end{array}\right)=-\left(\begin{array}{c} \alpha x_{1}x_{2}\\ \frac{r{\left(x_{2}+P_{2}\right)}^{2}}{k-c\left(x_{3}+G_{2}\right)}+\beta \left(x_{1}x_{2}\right)\\ \gamma_{1}x_{1}x_{3}+\gamma_{2}x_{2}x_{3} \end{array}\right)$$

From the above matrix, we derive the following quantities:

$$g_{20}^{0}=\frac{1}{4}\left\{\frac{\partial^{2}\eta_{1}}{\partial x_{1}^{2}}-\frac{\partial^{2}\eta_{1}}{\partial x_{2}^{2}}+2\frac{\partial^{2}\eta_{2}}{\partial x_{1}\partial x_{2}}+i\left(\frac{\partial^{2}\eta_{2}}{\partial x_{1}^{2}}-\frac{\partial^{2}\eta_{2}}{\partial x_{2}^{2}}-2\frac{\partial^{2}\eta_{1}}{\partial x_{1}\partial x_{2}}\right)\right\}=\frac{-1}{2}\left\{\beta +\left(\frac{r}{k-c\left(x_{3}+G_{2}\right)}-\alpha \right)i\right\},$$

$$g_{11}^{0}=\frac{1}{4}\left\{\frac{\partial^{2}\eta_{1}}{\partial x_{1}^{2}}+\frac{\partial^{2}\eta_{1}}{\partial x_{2}^{2}}+i\left(\frac{\partial^{2}\eta_{2}}{\partial x_{1}^{2}}+\frac{\partial^{2}\eta_{2}}{\partial x_{2}^{2}}\right)\right\}=\frac{-1}{2}\left\{\frac{r}{k-c\left(x_{3}+G_{2}\right)}i\right\},$$

$$G_{110}^{0}=\frac{1}{2}\left\{\frac{\partial^{2}\eta_{1}}{\partial x_{1}\partial x_{3}}+\frac{\partial^{2}\eta_{2}}{\partial x_{2}\partial x_{3}}+i\left(\frac{\partial^{2}\eta_{2}}{\partial x_{1}\partial x_{3}}-\frac{\partial^{2}\eta_{1}}{\partial x_{2}\partial x_{3}}\right)\right\}=-\left\{\frac{rc\left(x_{2}+P_{2}\right)}{{\left[k-c\left(x_{3}+G_{2}\right)\right]}^{2}}\right\},$$

$$G_{101}^{0}=\frac{1}{2}\left\{\frac{\partial^{2}\eta_{1}}{\partial x_{1}\partial x_{3}}-\frac{\partial^{2}\eta_{2}}{\partial x_{2}\partial x_{3}}+i\left(\frac{\partial^{2}\eta_{2}}{\partial x_{1}\partial x_{3}}+\frac{\partial^{2}\eta_{1}}{\partial x_{2}\partial x_{3}}\right)\right\}=\left\{\frac{rc\left(x_{2}+P_{2}\right)}{{\left[k-c\left(x_{3}+G_{2}\right)\right]}^{2}}\right\},$$

$$W_{11}^{0}=-\frac{1}{4\lambda_{3}(a_{1}\left(k^{*}\right)}\left(\frac{\partial^{2}\eta_{3}}{\partial x_{1}^{2}}+\frac{\partial^{2}\eta_{3}}{\partial x_{2}^{2}}\right)=0,$$

$$W_{20}^{0}=-\frac{1}{4\lambda_{3}(a_{1}\left(k^{*}\right)}\left(\frac{\partial^{2}\eta_{3}}{\partial x_{1}^{2}}+\frac{\partial^{2}\eta_{3}}{\partial x_{2}^{2}}-2i\frac{\partial^{2}\eta_{3}}{\partial x_{1}\partial x_{2}}\right)=0,$$

$$G_{21}^{0}=\frac{1}{8}\left\{\frac{\partial^{3}\eta_{1}}{\partial x_{1}^{3}}+\frac{\partial^{3}\eta_{1}}{\partial x_{1}\partial x_{2}^{2}}+\frac{\partial^{3}\eta_{2}}{\partial x_{2}^{3}}+\frac{\partial^{3}\eta_{2}}{\partial x_{1}^{2}\partial x_{2}}+i\left(\frac{\partial^{3}\eta_{2}}{\partial x_{1}^{3}}+\frac{\partial^{3}\eta_{2}}{\partial x_{1}\partial x_{2}^{2}}-\frac{\partial^{3}\eta_{1}}{\partial x_{2}^{3}}-\frac{\partial^{3}\eta_{1}}{\partial x_{1}^{2}\partial x_{2}}\right)\right\}=0,$$

The coefficient of the curvature of the limit cycle of the DPG system is

$$\sigma_{1}^{0}=Re\left\{\frac{g_{20}^{0}g_{11}^{0}}{4}i+G_{110}^{0}W_{11}^{0}+\frac{G_{21}^{0}+G_{101}^{0}W_{20}^{0}}{2}\right\},$$

$$\sigma_{1}^{0}=Re\left\{\frac{1}{16}\left(-\frac{\beta r}{\left(k-c\left(x_{3}+G_{2}\right)\right)}-\frac{r^{2}i}{\left(k-c(x_{3}+G_{2}\right)){}^{2}}+\frac{r\alpha i}{k-c\left(x_{3}+G_{2}\right)}\right)\right\}=\frac{-\beta r}{16\left(k-c\left(x_{3}+G_{2}\right)\right)}.$$

Thus, $\sigma_{1}^{0}<0$ provided condition (13) is satisfied; therefore, the DPG model has a stable limit cycle.

### Numerical simulation and discussion

**Fig. 2:**
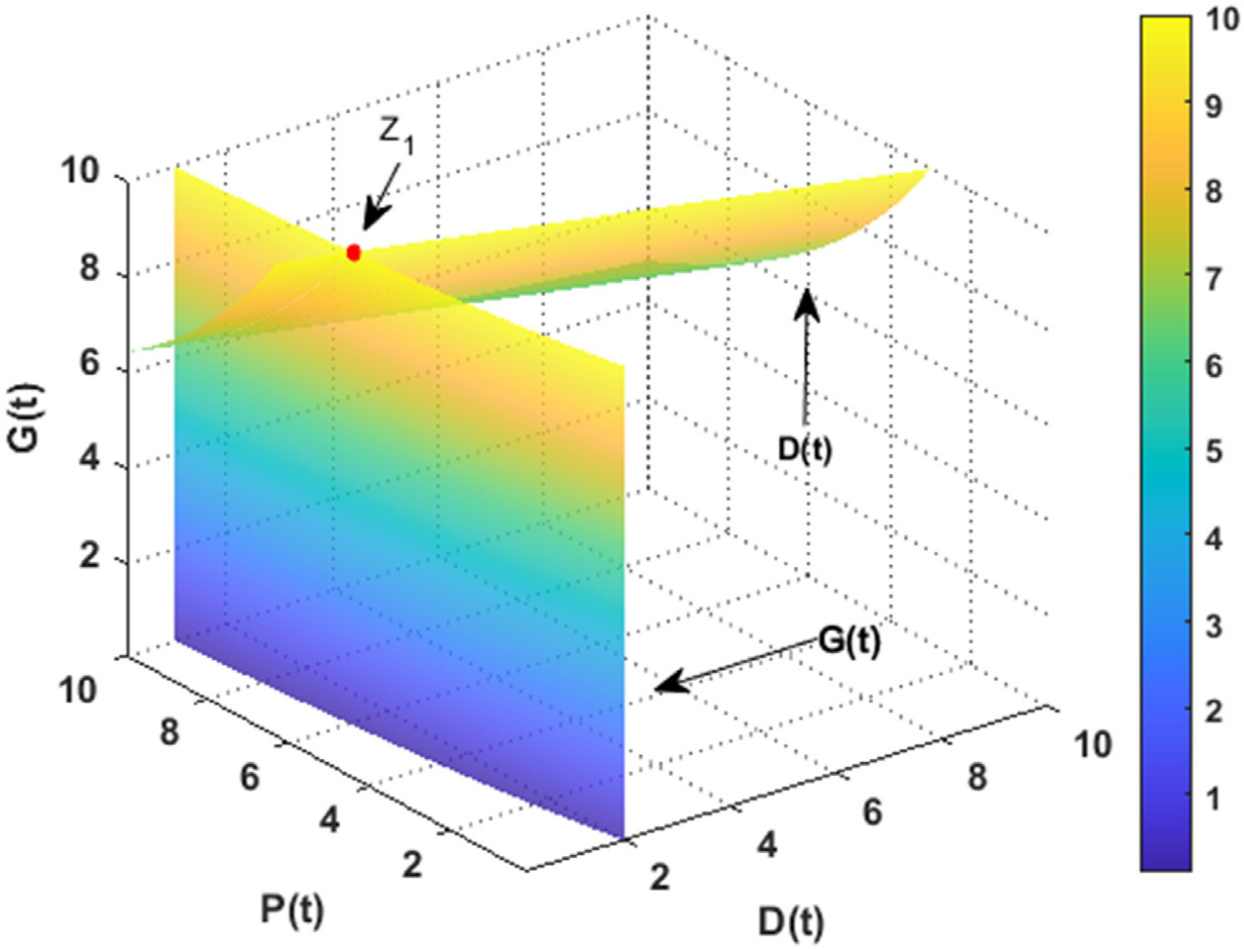
The coexistence of the desertification point with the data given in Table 1 when r=0.1.

**Fig. 3:**
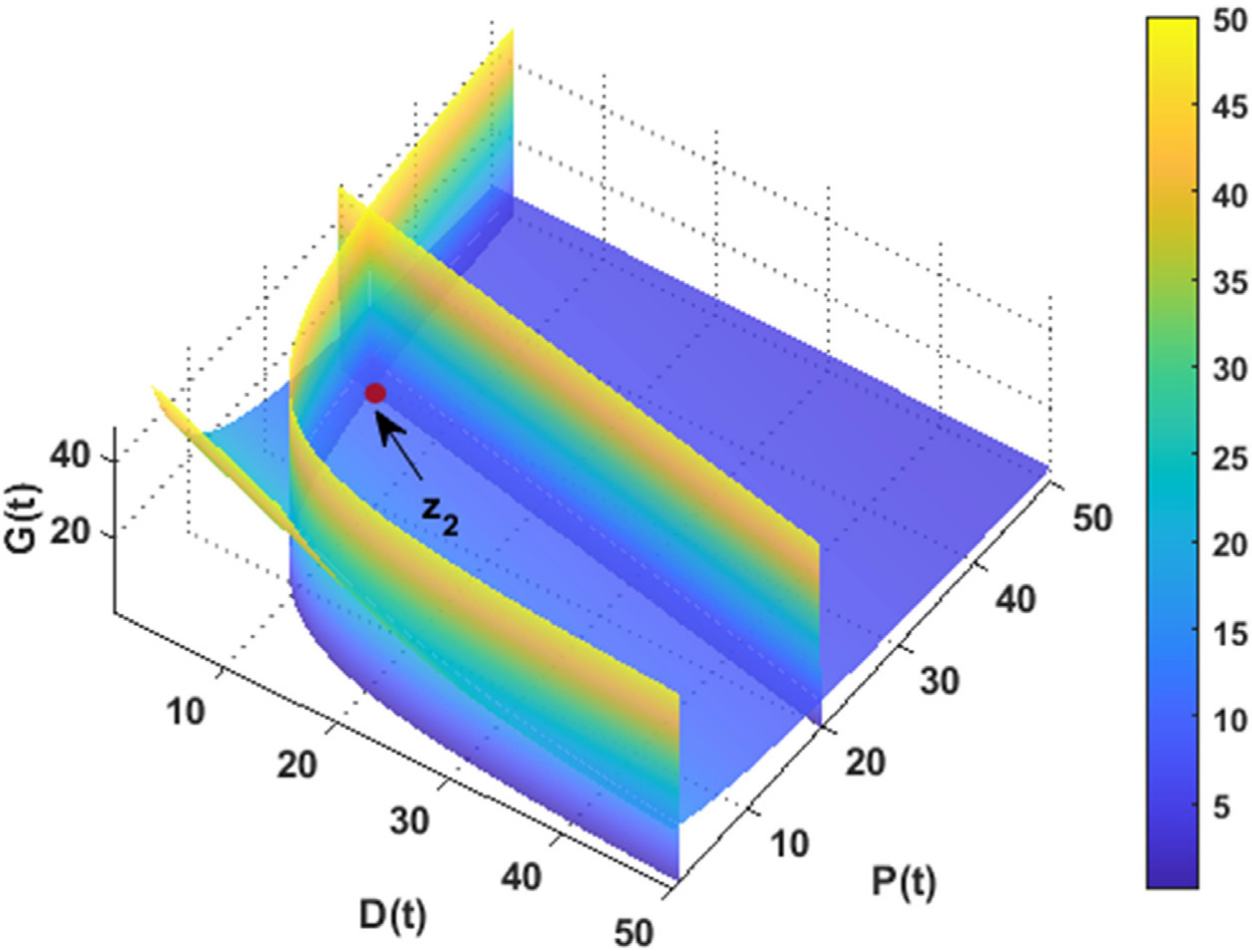
The coexistence of the non-desertification point with the data given in Table 1.

In this section, the dynamics of the DPG model are explored numerically using MATLAB. The simulations are conducted using data specified in Table 1. Figs. 2 and 3 illustrate the existence of the desertification and non-desertification equilibriums, respectively.

**Fig. 4:**
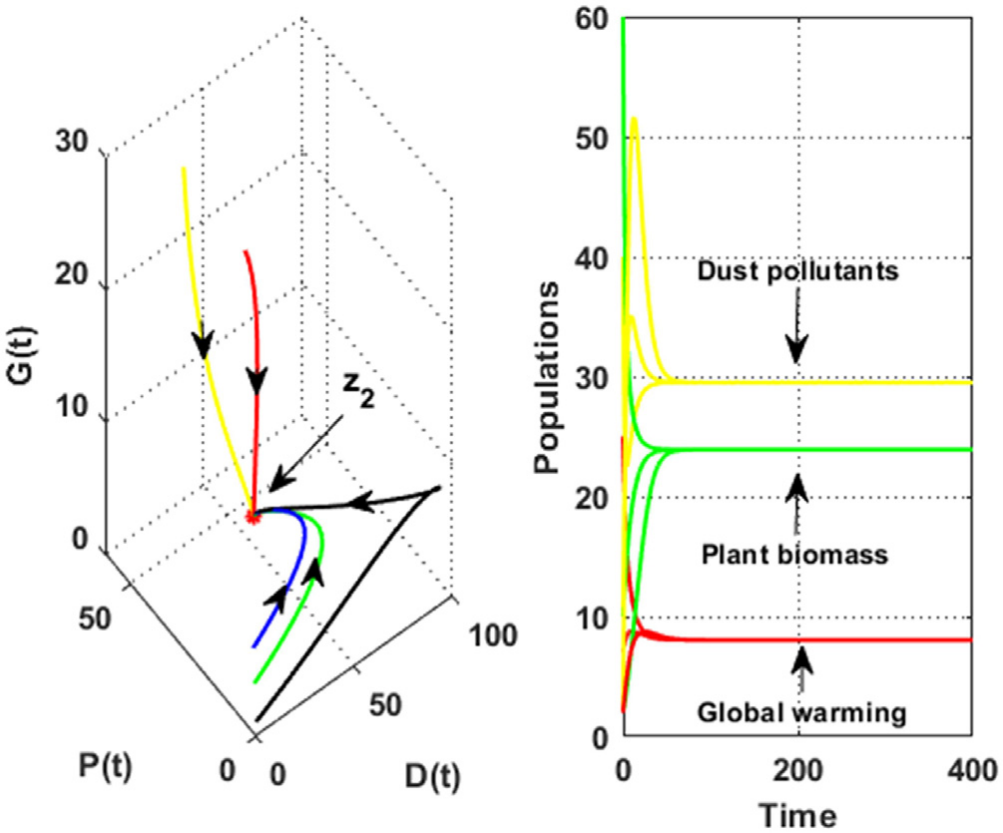
The dynamics of the DPG model with the data given in Table 1.

**Fig. 5:**
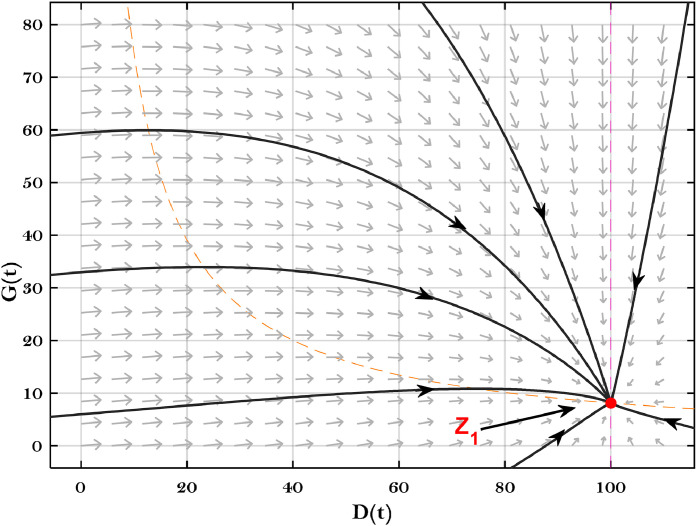
The global stability of the desertification equilibrium.

Further, Fig. 4 was generated by employing the parametric values in Table 1. This Figure indicates that dust pollutants, plant biomass and global warming are edging toward the non-desertification equilibrium $Z_{2}=\left(D_{2},\;P_{2},\;G_{2}\right)=\left(29.51,\;23.91,\;8.07\right).$ Moreover, the solution approaches the non-desertification equilibrium asymptotically despite the initial values. This behaviour shows that the global stability conditions stated in Theorem 4 have been satisfied. On the other hand, Fig. 5 shows the global stability of the desertification equilibrium $Z_{1}=\left(D_{1},\;0,\;G_{1}\right)=\left(99.51,\;0,\;9.01\right)$ in the absence of vegetation cover. This behaviour indicates that the global stability criteria outlined in Theorem 3 have been fulfilled.

**Fig. 6:**
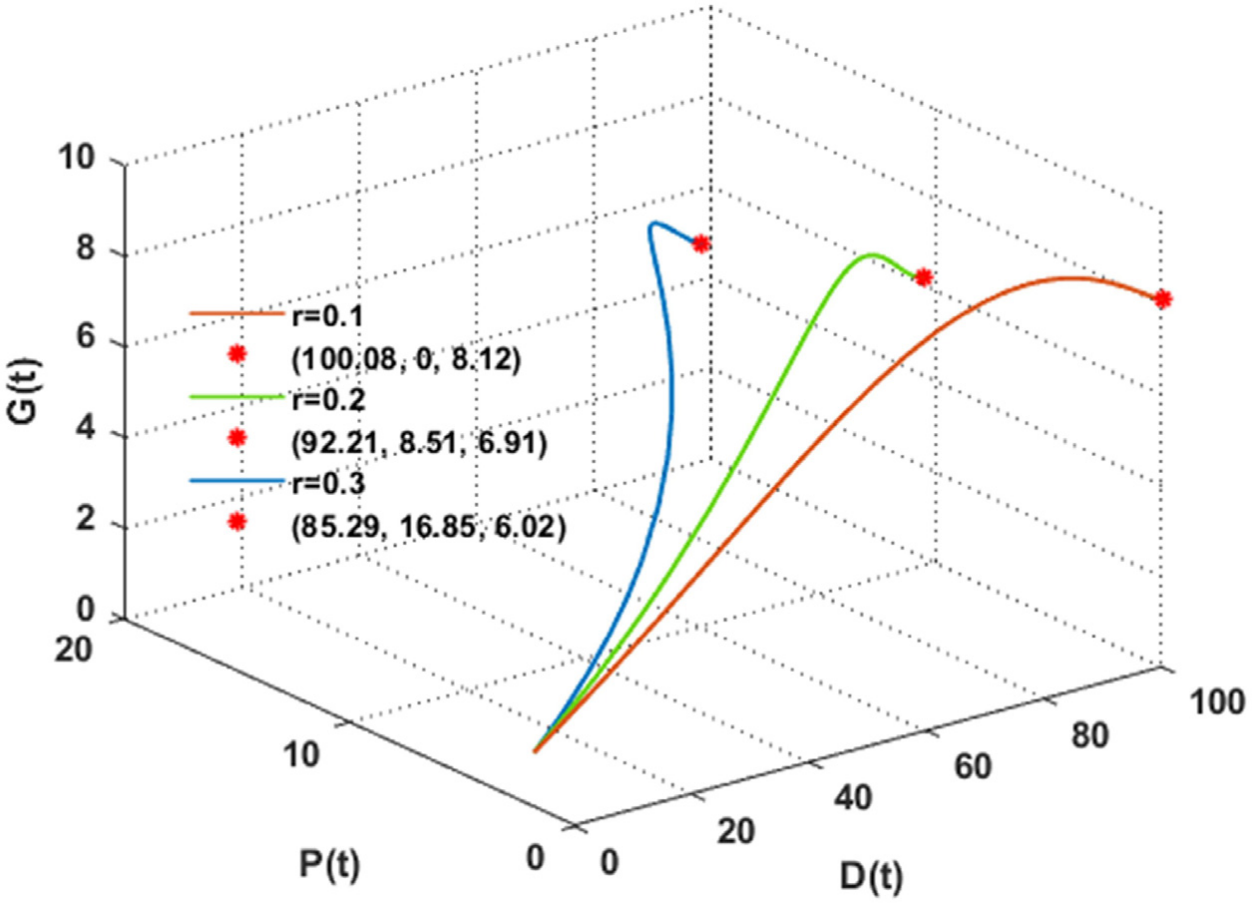
The dynamics of the DPG model with different values of r.

**Fig. 7:**
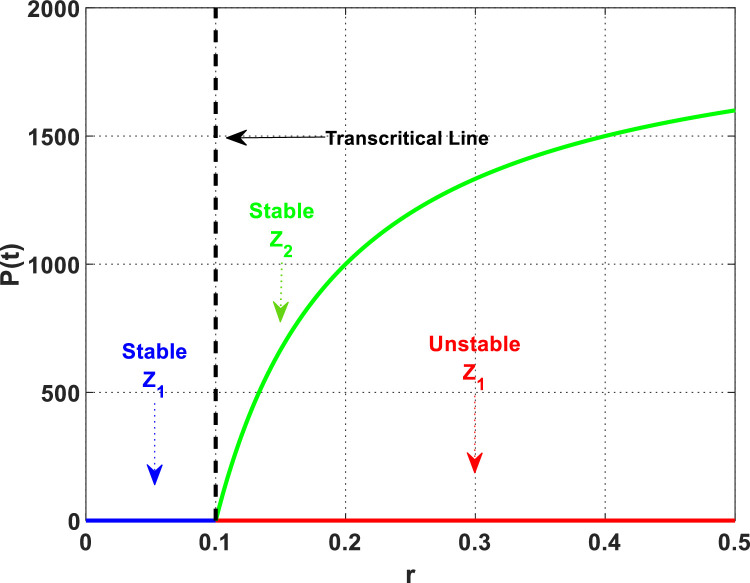
Transcritical bifurcation with respect to r.

The solution of the DPG system has been plotted in Fig. 6, Fig. 7, reflecting changes in the intrinsic growth rate r of plant biomass. The Figure indicates that an increase in r i,e, when r>0.1, corresponds with a gradual decrease in dust pollutants and global warming. Therefore, it can be concluded that an increase in plant plantations may help control dust pollutants and global warming. On the contrary, it could be observed that desertification is exacerbated by global warming and dust pollutants, which are exacerbated by the decrease in plant growth rate when r≤0.1. Plants are crucial in preventing soil erosion, as their roots stabilize the soil, avoiding the desertification resulting from erosion. Additionally, plants contribute to the preservation of soil moisture and the regulation of environmental temperature through processes such as transpiration and evaporation. The plant biomass cover deteriorates due to the soil becoming dryer as temperatures increase due to global warming, and apertures on the plant leaves' surface are sealed, hindering the plants' breathing process due to dust pollutants. As a result, the soil becomes exposed and susceptible to erosion by wind and precipitation as the cover diminishes, which leads to desertification. It is clear from Fig. 6 that for a small value of r=0.1; the DPG system settles down asymptotically to the desertification equilibrium $Z_{1}=\left(100.08,\;0,\;8.12\right)$. Moreover, if we raise the value of r, i.e. (say r=0.2), we observe that the DPG system approaches asymptotically to the non-desertification equilibrium $Z_{2}=\left(92.12,\;8.51,\;6.91\right)$. Therefore, Theorem 6 is satisfied, and the DPG system faces a transcritical bifurcation at r=0.1. See Fig. 6.

**Fig. 8:**
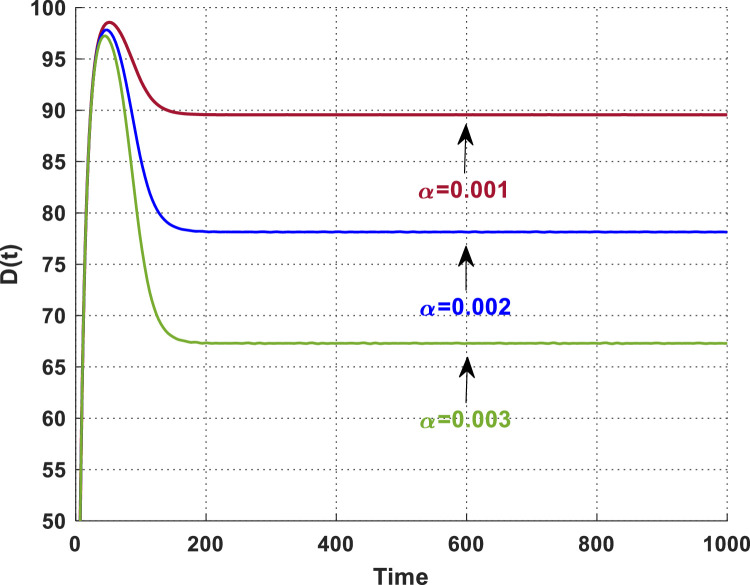
The dynamics of the concentration of dust pollutants with different values of α.

The temporal change of dust pollutant concentration for various values of α is illustrated in Fig. 8. The statistics clearly indicate that when the interaction rate of dust pollutants with plant biomass increases, the concentration of dust pollutants diminishes.

**Fig. 9:**
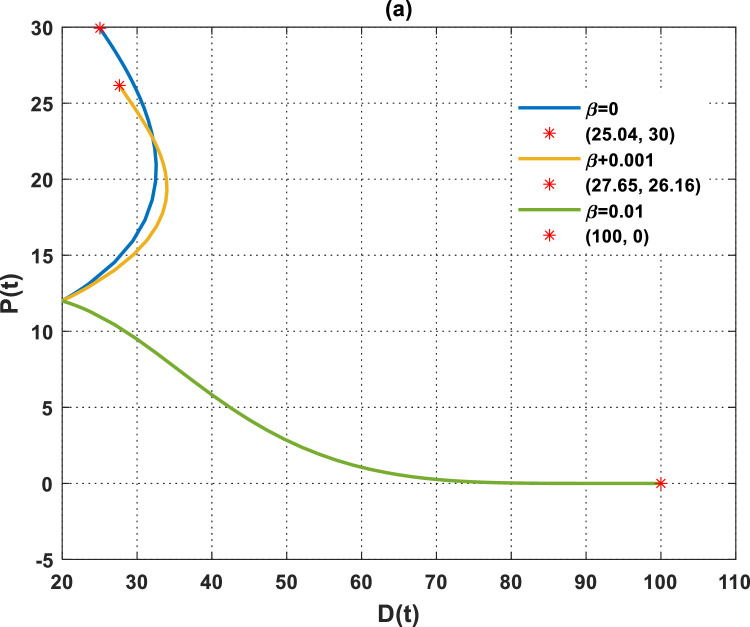
The dynamics of the DPG model with different values of β.

Fig. 9 demonstrates the relationship between plant biomass density and dust pollutant concentration across different β values. The Figure indicates that an increase in the depletion rate coefficient of plant biomass due to dust pollutants is associated with a rise in atmospheric dust pollutant concentration and a decrease in plant biomass density. The density of plant biomass reaches its maximum, corresponding to carrying capacity, in the absence of dust pollutants (i.e., when (β=0), leading to the minimum equilibrium concentration of dust pollutants (Fig. 9). The augmented plant biomass density in the greenbelt, which remains unaffected by dust pollutants, will effectively decrease the concentration of dust pollutants in the atmosphere. Conversely, an increase in beta leads to a rapid increase in the concentration of dust pollutants, as observed when β=0.01. This negatively affects vegetation, resulting in the desertification of green areas, and the solution of the DPG model will stabilize at the desertification equilibrium.

**Fig. 10:**
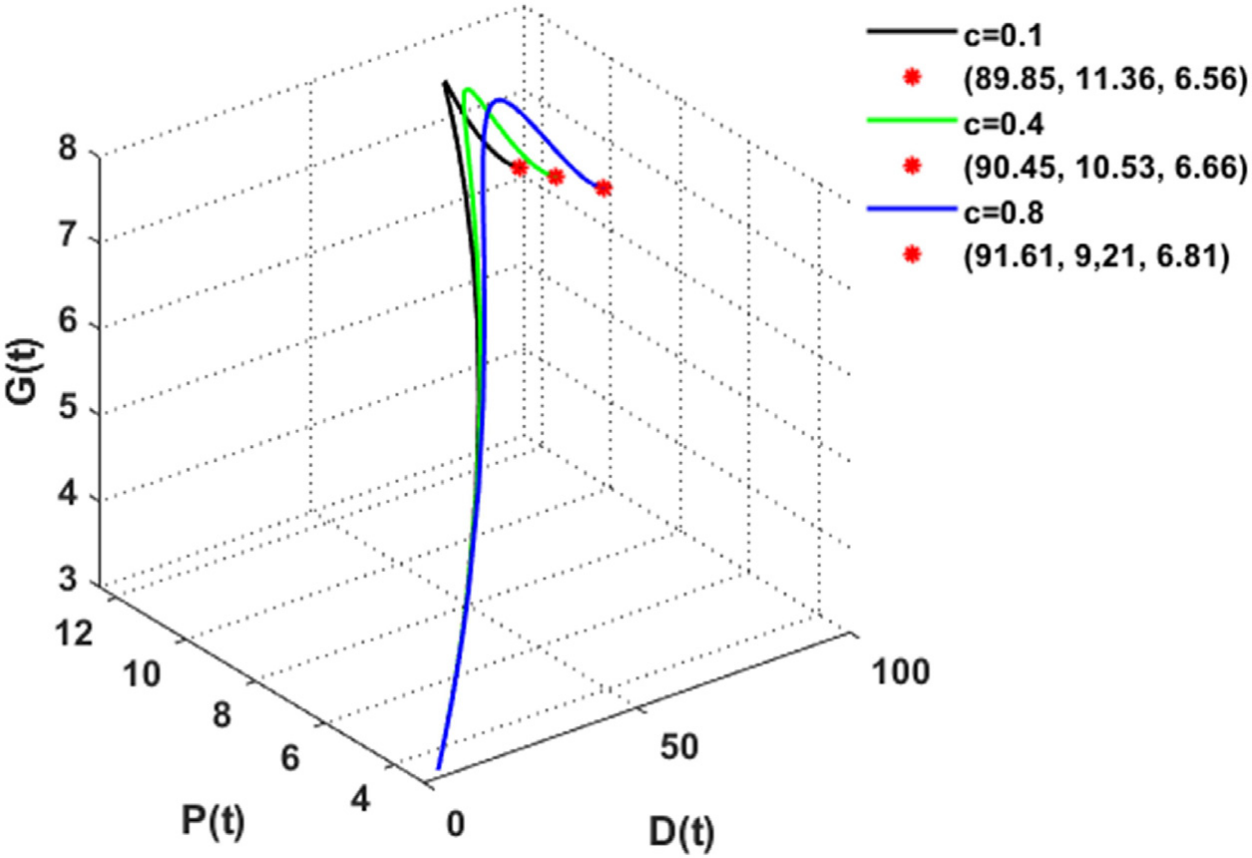
The dynamics of the DPG model with different values of c.

The effect of the rising global warming-induced desertification rate c on the decrease of vegetation cover is depicted in Fig. 10. It is observed that increasing global warming can limit plant growth since rising temperatures harm the vegetation cover in verdant areas. As temperatures increase and arid periods prolong, plants become more vulnerable to drought. This causes land deterioration and the progressive transformation of these areas into deserts.

**Fig. 11:**
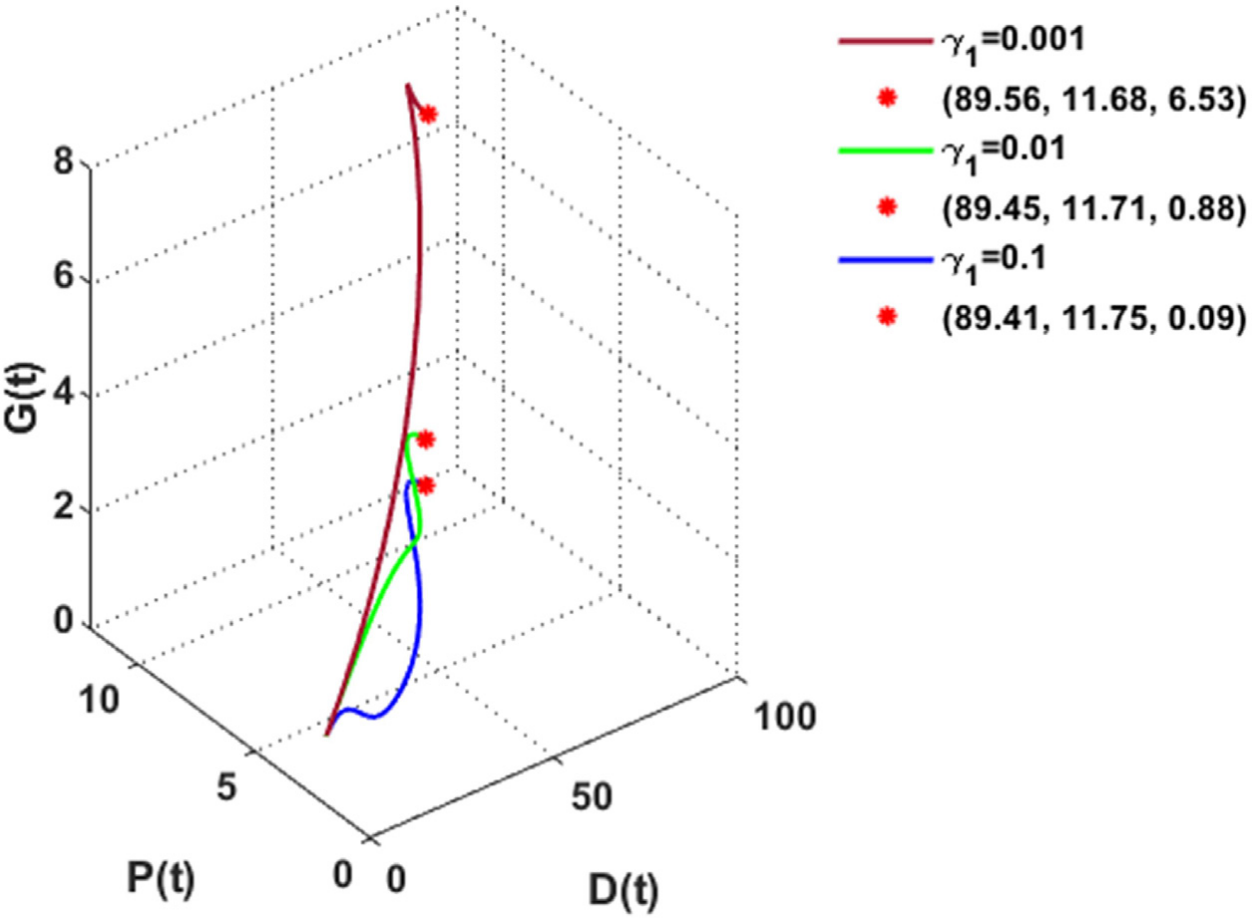
The dynamics of the DPG model with different values of $\gamma_{1}$.

**Fig. 12:**
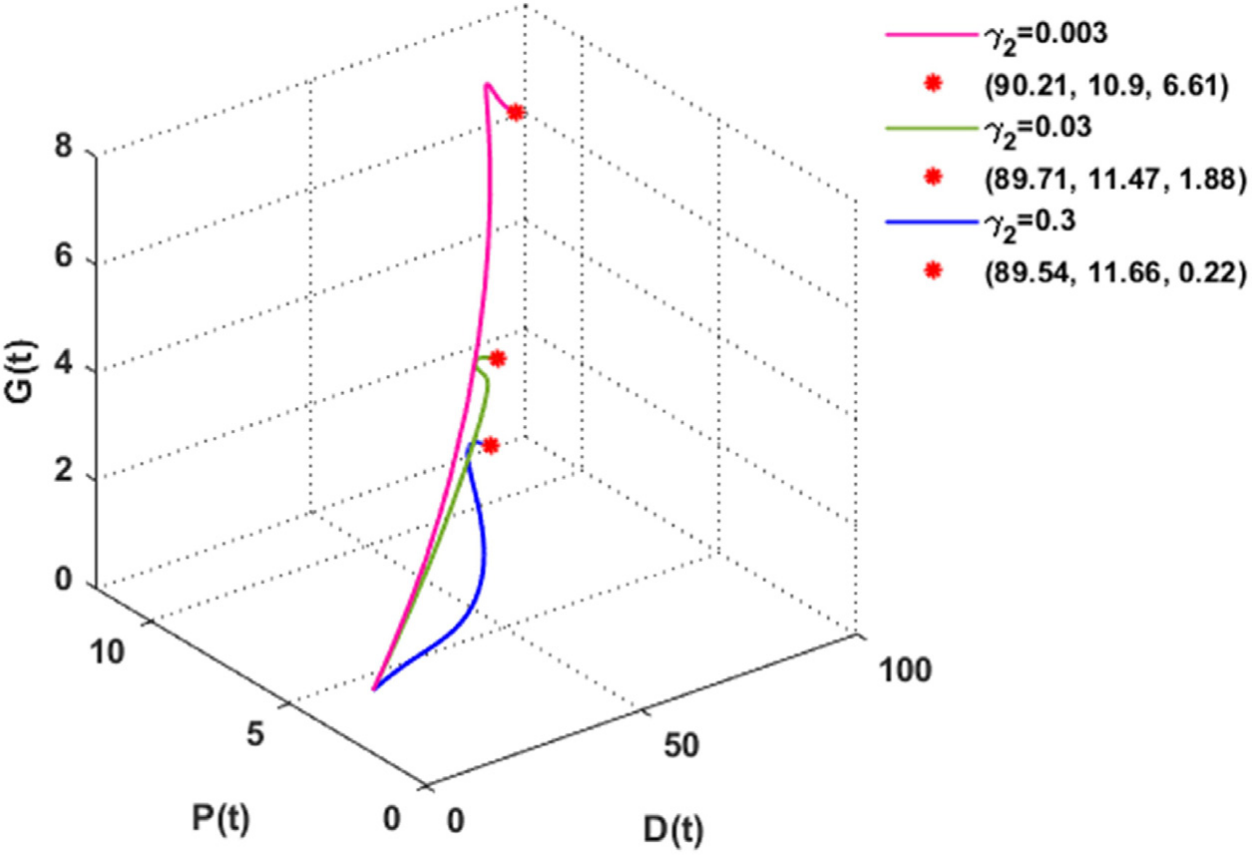
The dynamics of the DPG model with different values of $\gamma_{2}$.

Figs. 11 and 12 consider different values of $\gamma_{1}$ and $\gamma_{2}$ i.e., the depletion rate of global warming due to dust pollutants and plant biomass, respectively. These figures show that plant biomass increases gradually with the rising depletion rates of global warming.

**Fig. 13:**
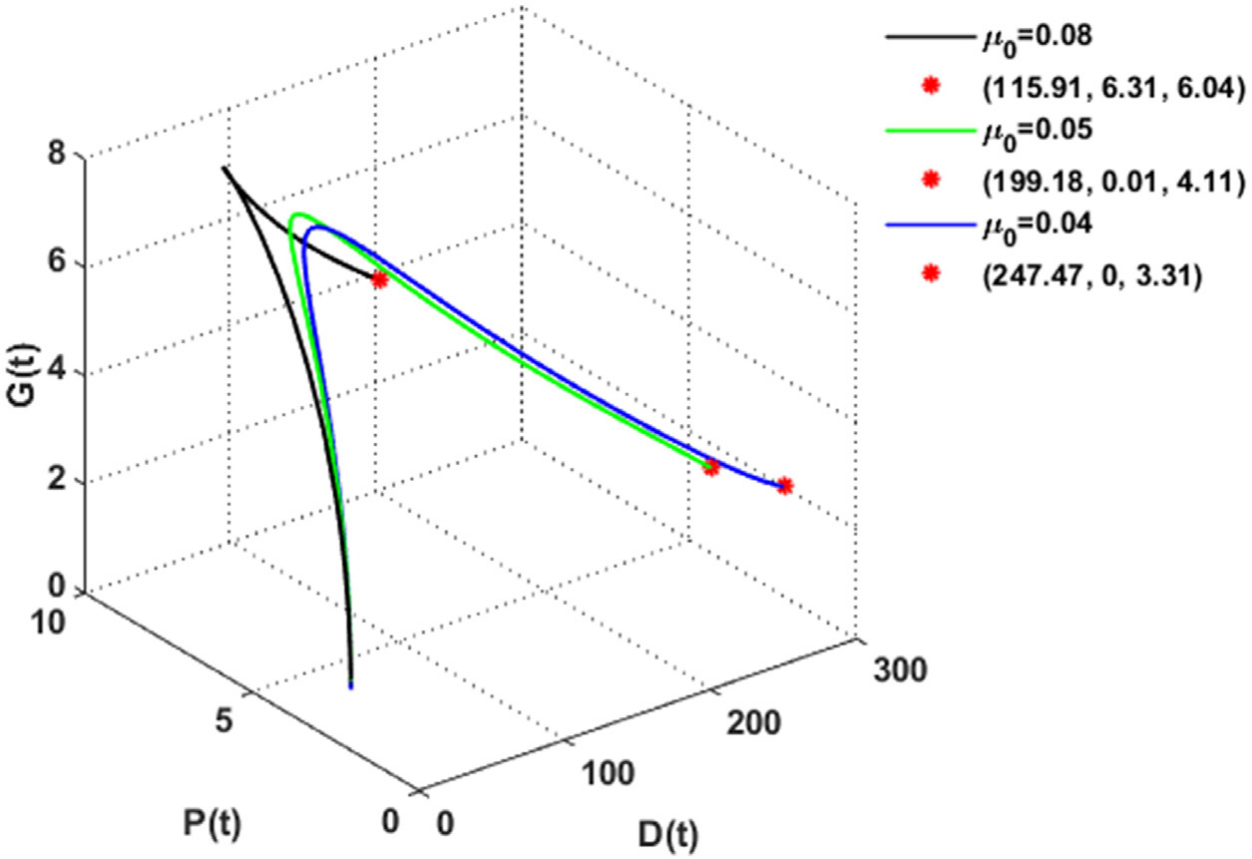
The dynamics of the DPG model with different values of $\mu_{0}$.

Finally, changes in the coefficient of natural depletion of dust pollutants, i.e., $\mu_{0}$ is drawn in Fig. 13. It is seen that the decrease in $\mu_{0}$, causes a gradual increase in the concentration of dust particles. Further, vegetation will be adversely affected by an increase in dust, which may result in the mortality or deterioration of plants in certain instances. The consequences consist of obstruction of sunlight: Dust deposition on plant leaves reduces the amount of light that reaches them, affecting the process of photosynthesis, which is the foundation of plant growth. Further, clogged pores: Plant leaves possess pores that are employed to absorb carbon dioxide and release oxygen. The gas exchange essential for growth can be impeded by dust obstructing these apertures. Consequently, vegetation may be adversely affected in environments where dust accumulates substantially and continuously, resulting in its deterioration. In addition, it can be realized that the solution for the DPG system is stabilized at the desertification equilibrium $Z_{1}=\left(247.47,\;0,\;3.31\right)$ when $\mu_{0}=0.04$.

**Fig. 14:**
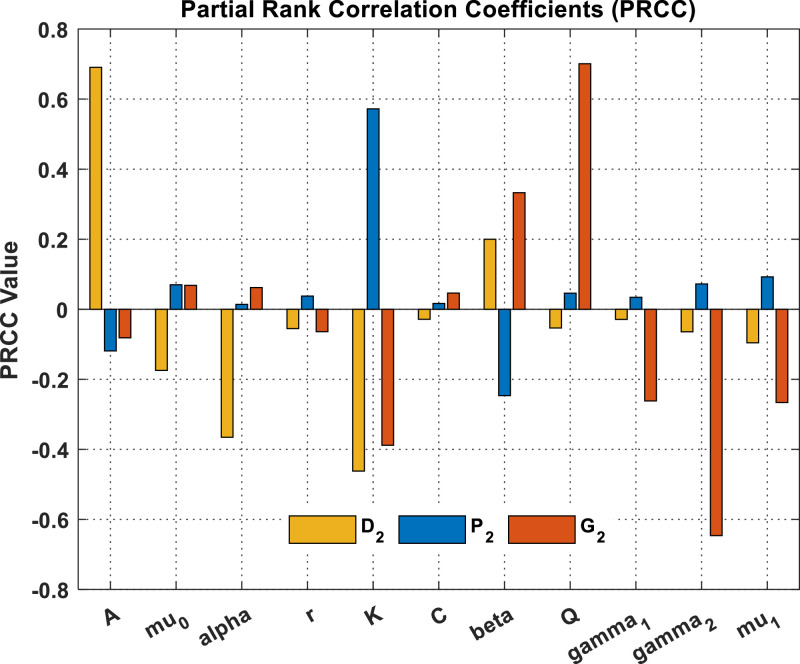
The sensitivity of parameters relative to the DPG system for the non-desertification point.

On the other hand, equilibrium points signify the steady or long-term behaviour a system may achieve under particular conditions. Consequently, sensitivity analysis of equilibrium points in dynamic systems has substantial importance in research. It aids in comprehending the variations in the system's behaviour and stability under diverse parameter settings. By examining the system's reaction to parameter fluctuations, we may ascertain the critical parameters that exert the greatest influence on system performance, allowing their optimization to improve performance and stability. We employ partial rank correlation coefficients (PRCCs) to examine the sensitivity of the DPG system coexistence equilibrium points. The parameters $A,\;\alpha ,\;\beta ,\;r,\;k,\;c,\;Q,\;\mu_{0},\;\mu_{1},\;\gamma_{1}$ and $\gamma_{2}$ serve as input parameters, while the output variables $D_{2},\;P_{2},\;$and $G_{2}$ are determined through system (1). Subsequently, using the parameter set in Table 1, we generate Fig. 14. Fig. 14 indicates that dust pollutants exhibit heightened sensitivity to the resource input of dust pollutants from diverse sources, i.e., A, which strongly influences $D_{2}$. Whereas k and α significantly reduce dust pollutants. Further, global warming is strongly affected by resource input that is rising global warming, i.e., Q, while $\gamma_{2}$ and k have a big role in reducing global warming. It could be concluded that the carrying capacity of the plant biomass is a key parameter that affects the coexistence of the non-desertification point $Z_{2}=\left(D_{2},\;P_{2},\;G_{2}\right)$.

## Conclusions

Dust pollutants and global warming significantly endanger the environment. This may result in the progressive transformation of green spaces into unsustainable areas. This paper introduces and evaluates a mathematical model that investigates the impact of global warming and dust contaminants on plant biomass growth. The stability theory of differential equations is employed to conduct the model analysis. The model analysis indicates that the system has two equilibrium points: non-desertification equilibrium and desertification equilibrium. Some intriguing findings regarding equilibrium points' stability are presented in the model analysis. The model analysis yields intriguing findings regarding certain types of bifurcations, including transcritical and Hopf bifurcation around the equilibrium points. In addition, the following results were observed from the numerical simulation:

1. The concentration of dust pollutants in the atmosphere decreases as the interaction rate between dust pollutants and plant biomass increases.
2. The plant biomass faces the danger of transferring the green space into desertification if the intrinsic growth rate of plant biomass, the dust pollutants-induced plant biomass depletion coefficient and the coefficient of natural depletion of dust pollutants cannot be controlled.
3. It could avoid desertification if the conditions stated in Theorem 4 guarantee that plant biomass can coexist with global warming and dust pollutants in a stable state.
4. From the sensitivity analysis of the non-desertification equilibrium point, the results show that the carrying capacity of plant biomass is a critical parameter which plays a significant role in decreasing the negative impact of dust pollutants and global warming. Thus, increasing vegetation through the following reforestation policy is crucial in preventing desertification and influencing coexistence at the non-desertification point.

In the future, we will investigate how to expand the model to incorporate interactions with other ecological factors, such as animal populations, particularly those that consume plants.

## Limitations

Not Applicable.

## Ethics authors statements

The platforms' data redistribution policies were complied with.

## Funding statement

This research received no external funding.

## Supplementary material and/or additional information

Not applicable.

## CRediT authorship contribution statement

**Eman Hakeem:** Conceptualization, Writing – original draft. **Shireen Jawad:** Conceptualization, Methodology, Writing – review & editing. **Ali Hasan Ali:** Methodology, Project administration, Software, Validation, Writing – review & editing. **Mohamed Kallel:** Data curation, Investigation. **Husam A. Neamah:** Formal analysis, Resources.

## Declaration of competing interest

The authors declare that they have no known competing financial interests or personal relationships that could have appeared to influence the work reported in this paper.

## Data Availability

Data will be made available on request.
